# Role of Bcl2-associated Athanogene 3 in Turnover of Gap Junction Protein, Connexin 43, in Neonatal Cardiomyocytes

**DOI:** 10.1038/s41598-019-44139-w

**Published:** 2019-05-21

**Authors:** Farzaneh Ghasemi Tahrir, Manish Gupta, Valerie Myers, Jennifer Gordon, Joseph Y. Cheung, Arthur M. Feldman, Kamel Khalili

**Affiliations:** 1 Department of Neuroscience, Center for Neurovirology, Lewis Katz School of Medicine at Temple University, Philadelphia, Pennsylvania USA; 2 Department of Medicine, Lewis Katz School of Medicine at Temple University, Philadelphia, Pennsylvania USA; 3 Cardiovascular Research Center, Lewis Katz School of Medicine at Temple University, Philadelphia, Pennsylvania USA; 4 Center for Translational Medicine, Lewis Katz School of Medicine at Temple University, Philadelphia, Pennsylvania USA

**Keywords:** Molecular biology, Cardiology

## Abstract

Any pathological stress that impairs expression, turnover and phosphorylation of connexin 43 (Cx43), one of the major proteins of gap junctions, can adversely impact myocardial cell behavior, thus leading to the development of cardiac arrhythmias and heart failure. Our results in primary neonatal rat ventricular cardiomyocytes (NRVCs) show that impairment of the autophagy-lysosome pathway dysregulates degradation of Cx43, either by inhibiting lysosomal activity or suppressing the level of Bcl2-associated athanogene 3 (BAG3), a stress-induced pleiotropic protein that is involved in protein quality control (PQC) via the autophagy pathway. Inhibition of lysosomal activity leads to the accumulation of Cx43 aggregates and suppression of BAG3 significantly diminished turnover of Cx43. In addition, knock-down of BAG3 reduced the levels of Cx43 by dysregulating Cx43 protein stability. Under stress conditions, expression of BAG3 affected the state of Cx43 phosphorylation and its degradation. Furthermore, we found that BAG3 co-localized with the cytoskeleton protein, α-Tubulin, and depolymerization of α-Tubulin led to the intracellular accumulation of Cx43. These observations ascribe a novel function for BAG3 that involves control of Cx43 turnover under normal and stress conditions and potentially for optimizing communication of cardiac muscle cells through gap junctions.

## Introduction

Gap junctions are protein structures that permit metabolic and electrical coupling between two neighboring cells and mediate cell-to-cell communication. Each gap junction channel consists of two hemi-channels on the plasma membrane of two adjacent cells known as connexons. Connexons are formed by the assembly of hexameric complexes of connexin transmembrane proteins^1^. In the healthy heart, conduction of action potential through gap junctions maintains regular rhythm and contraction; while under pathological conditions, impairment of action potential propagation throughout the myocardium results in arrhythmia and cardiovascular disease^2^. Connexin 43 (Cx43), a member of the connexin family of proteins, is highly expressed in heart tissue and has a short half-life of between 1–5 hours^3^. Therefore, maintaining homeostasis and turnover of Cx43 in plasma membrane is of great importance for maintaining conduction of cardiac tissue and avoiding arrhythmia^3,4^.

Previous reports have described degradation of Cx43 through three main pathways: autophagy^4^, ubiquitin-proteasome^5^, and endo-lysosome^6^ pathways. Autophagy is an intracellular degradation mechanism that plays an important role in maintaining cardiac homeostasis by regulating cellular metabolism and energy balance^7^. In this process, damaged organelles and protein aggregates are engulfed within double-membrane vesicles, referred to as autophagosomes, and delivered for degradation and removal by lysosomes^8^. The protective role of autophagy in the heart under stress conditions has been reported by several laboratories^9–11^. Recently, we reported that induction of autophagy in mice with myocardial ischemia/reperfusion injury significantly reduced infarct size and ameliorated heart dysfunction^9^. Earlier studies demonstrated that autophagy is also involved in the degradation of internalized gap junctions in cells overexpressing Cx43^12^. However, the underlying mechanisms and key molecular players are poorly understood.

BAG3 is a 575-amino acid protein that is highly expressed in cardiac and skeletal muscle^13^. BAG3 interacts with the ATPase domain of heat shock protein 70 (HSP70) through its BAG domain and regulates the function of the HSP70/HSC70 molecular chaperone to maintain protein homeostasis^14^. Among the various regulators of PQC, BAG3 has been reported to be a key regulator of the autophagy pathway in various cell types including the heart. BAG3 functions as an anti-apoptosis protein through binding with Bcl-2 and overexpression of BAG3 in cancer cells contributes to resistance to chemotherapy^15^. Mutations of BAG3 impair Z-disc assembly and lead to the development of dilated cardiomyopathy^16–18^. BAG3 deficiency results in myofibrillar degeneration followed by the development of lethal cardiomyopathy and death by 4 weeks of age^19^. While the role of BAG3 in quality control has been reported by several researchers, to our knowledge, there is no study describing the impact of BAG3 on gap junction turnover, either in basal or stress conditions. We hypothesized that BAG3 might be an important molecular player in regulating the life cycle of Cx43 and evaluated the role of BAG3 in maintaining Cx43 homeostasis.

In this study, we used primary cultures of NRVCs and found that there was a significant reduction in Cx43 abundance as well as Cx43 turnover in BAG3-suppressed cardiomyocytes. In addition, we found that suppression of BAG3 resulted in destabilization of Cx43. We also examined the impact of BAG3 on Cx43 degradation under starvation stress conditions and found that when BAG3 was knocked down, stress-induced degradation of Cx43 was significantly impaired. In addition, our results showed that BAG3 colocalized with the tubulin network and tubulin depolymerization led to significant accumulation of Cx43 within cardiomyocytes.

## Results

### Knock-down of BAG3 reduces lysosomal turnover and protein levels of Cx43

**Figure 1:**
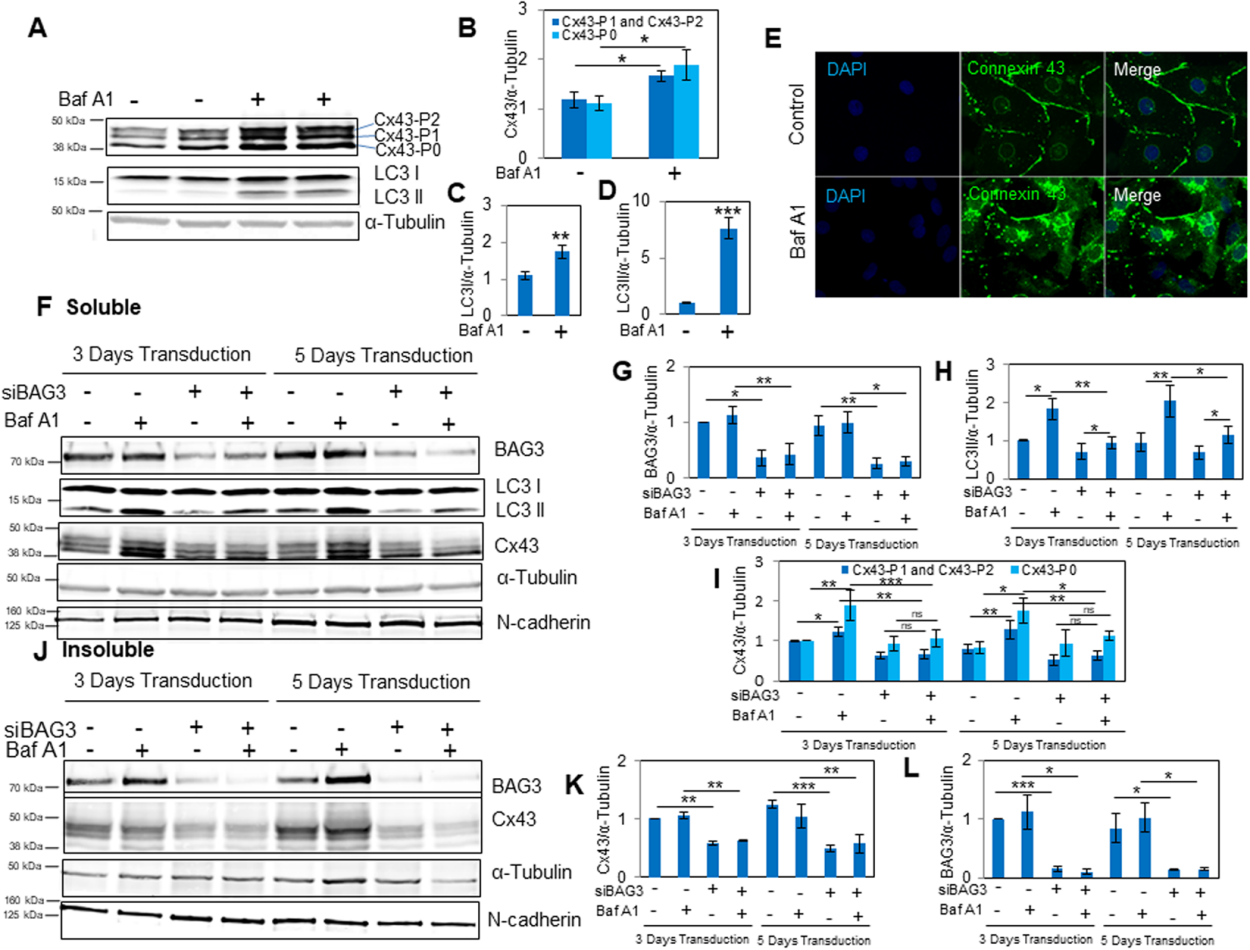
Cx43 undergoes lysosome degradation and knock-down of BAG3 reduces turnover of Cx43. (**A**) NRVCs were treated with the lysosomal inhibitor BafA1 (50 nM, 3 hours) and the levels of autophagy proteins, LC3 I and LC3 II, and Cx43 were evaluated using Western blot analysis. (**B**–**D**) The levels of LC3 I, LC3 II, Cx43-P0, Cx43-P1 and Cx43-P2 were quantified based on the data represented in (A) (n = 4). (**E**) NRVCS were treated either with DMSO as control cells or Baf A1 (50 nM, 3 hours), fixed with 4% PFA and stained with an antibody against Cx43. Microscopic imaging indicated that Cx43 was highly expressed in plasma membrane of control NRVCs. Internalized Cx43 aggregates were observed in NRVCs treated with lysosomal inhibitor BafA1. (**F**–**I**) NRVCs were transduced with either Ad-siBAG3 or Ad-control for 3 or 5 days in the presence or absence of lysosomal inhibitor BafA1 (50 nM, 3 hours). Lysis buffer-soluble protein fractions of the cells were extracted and the levels of BAG3, LC3 II, Cx43-P0, Cx43-P1 and Cx43-P2 were evaluated by Western blot and quantified. Lysosomal turnover of LC3 II, Cx43-P0, Cx43-P1 and Cx43-P2 were significantly inhibited in NRVCs knocked down for BAG3 (n = 3). (**J**–**L**) NRVCs were transduced with either Ad-siBAG3 or Ad-control for 3 or 5 days in the presence or absence of BafA1 (50 nM, 3 hours). Lysis buffer-insoluble protein fractions of cell lysates were extracted and BAG3 and Cx43 levels were measured and quantified. Western blot and quantifications indicated that Cx43 protein levels were significantly reduced in NRVCs knocked down for BAG3 (n = 3). N-cadherin was detected in both soluble and insoluble fractions as a plasma membrane marker. α-tubulin served as a loading control for both soluble and insoluble protein fractions. *p < 0.05; **p < 0.01; ***p < 0.001.

As a first step, expression of Cx43 was analyzed by Western blot in protein lysates of NRVCs. A triplet of bands were detected on SDS-PAGE representing slower-migrating forms of Cx43 (Cx43-P1 and Cx43-P2) and a faster-migrating form of Cx43 (Cx43-P0) around 40 kDa to 43 kDa, which are indicative of its post-translational modifications and phosphorylation state^20^. In order to investigate whether Cx43 degradation in NRVCs occurs through lysosomes, lysosomal activity was inhibited by treating the cardiomyocytes with 50 nM Bafilomycin A1 (BafA1) for 3 hours and the levels of the autophagy markers, LC3 I and LC3 II, and Cx43 were analyzed by Western blot. BafA1 inhibits lysosomal acidification and blocks the fusion between autophagosome and lysosome by impairing the function of proton pumps in the lysosomal membrane^21^. Data indicated that inhibition of lysosomes resulted in a significant increase in the levels of LC3 I and LC3 II. Western blot data also demonstrated that the levels of Cx43-P0, -P1 and –P2 significantly increased as a result of lysosomal blockage (Fig. 1A–D). Next, we evaluated the levels of Cx43 in soluble and insoluble protein fractions of cells treated with siRNA to knock down the lysosomal marker, Lamp 2 (Thermo Fisher Scientific). Western blot data indicated that Cx43 levels did not change in the soluble fraction (data not shown), while the level of Cx43 significantly increased in the insoluble fraction of cells with suppressed Lamp2 (Fig. S1A,B). These findings were further confirmed by monitoring the subcellular localization of Cx43 and cell morphology in cardiomyocytes treated with BafA1 versus control cells. Immunocytochemistry (ICC) indicated that Cx43 was highly expressed in the plasma membrane of primary cardiomyocytes. In the presence of BafA1, Cx43 aggregates accumulated in the cytoplasm; suggesting that Cx43 degrades through a lysosomal pathway (Fig. 1E). To further investigate the mechanisms underlying Cx43 degradation in NRVCs, cells were double labeled using antibodies against ubiquitin and Cx43. Results from microscopic imaging indicated the presence of Cx43 aggregates in the perinuclear area, which colocalized with ubiquitin (Fig. S1C). We also investigated whether Cx43 degrades through proteasomes in cardiomyocytes. For this purpose, cells were treated for 12 hours with 5 µM of the proteasome inhibitor MG132 and Cx43 levels were investigated. Western blot showed that only the levels of Cx43-P1 and Cx43–P2 were significantly increased as a result of proteasome inhibition (Fig. S1D,E). Immunocytochemistry also indicated that inhibition of the proteasome led to the accumulation of Cx43 aggregates in cardiomyocytes (Fig. S1F).

BAG3 binds to the HSP70/HSC70 chaperone complex to regulate degradation of misfolded and damaged proteins through the autophagy-lysosome pathway^22^. The role of BAG3 in maintaining PQC has been investigated by several laboratories but, to our knowledge, its role in the turnover of Cx43 has not been previously reported. To understand the role of BAG3 in the turnover of Cx43, BAG3 was knocked down in NRVCs using adenovirus transduction of BAG3 siRNA for 3 and 5 days. Transduced cardiomyocytes were then treated with BafA1 and the levels of the autophagy marker, LC3 II, and Cx43 were evaluated. Western blot of the soluble fractions showed that LC3 II levels were significantly reduced in BAG3-suppressed cardiomyocytes compared to control cells at 3 and 5 days post transduction. Furthermore, Cx43 (P0, P1 and P2) levels were significantly increased in the presence of BafA1 in control cells, but BafA1 treatment did not lead to a significant increase in Cx43 levels in NRVCs with BAG3 knock-down, suggesting that knock-down of BAG3 impaired lysosomal turnover of Cx43 (Fig. 1F–I). Since Cx43 is a membrane-spanning protein, we analyzed the insoluble fraction of the cell lysates as well. Western blot revealed that Cx43 protein levels were significantly reduced in NRVCs knocked down for BAG3 (Fig. 1J–L). Protein kinase C (PKC) activation leads to Cx43 phosphorylation on serine 368 (pSer368Cx43), which modulates its internalization and degradation^23^. ICC data indicated that pSer368Cx43 is only expressed in the plasma membrane of NRVCs and not in the perinuclear area (Fig. S1G). Further analysis of the insoluble cell fraction by Western blot indicated that BAG3 suppression significantly reduced the level of pSer368Cx43 in NRVCs (Fig. S1H,I). We also investigated the effect of BAG3 suppression on proteasomal degradation of Cx43. For this purpose, transduced cardiomyocytes were treated with MG132 (5 µM, 12 hours) and the levels of Cx43 were investigated. Western blot of the soluble and insoluble fractions indicated that Cx43 proteasomal degradation was not affected by BAG3 suppression (Fig. S1J–M). N-cadherin, which functions as a transmembrane adhesion protein, and α-tubulin were detected in both the soluble and insoluble fractions, with α-tubulin serving as a loading control for both fractions. Taken together, these results suggest that dysregulating the autophagy-lysosome pathway, either by inhibiting the activity of lysosomes or suppressing BAG3 as a key molecular player in autophagy, impairs proper turnover of Cx43.

### BAG3 plays an important role in regulating stability of Cx43 protein

**Figure 2:**
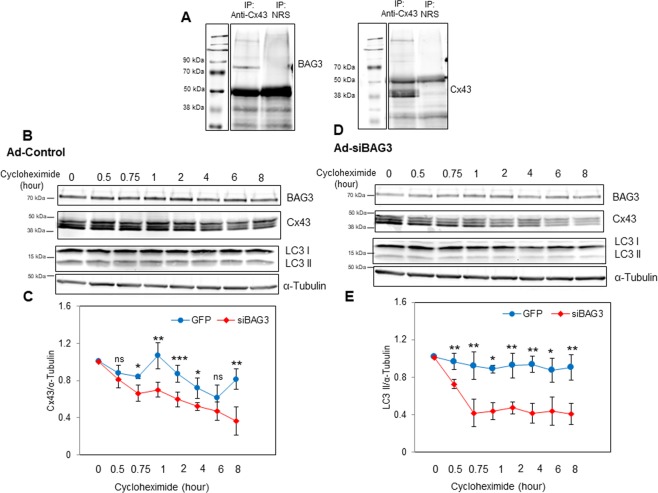
BAG3 regulates the stability of Cx43. (**A**) Co-immunoprecipitation was performed by using an antibody to pull down Cx43 and normal rabbit serum (NRS) in protein lysates of NRVCs. The protein components of the immunocomplexes were then analyzed by Western blot using antibodies against BAG3 and Cx43. (**B–E**) NRVCs were transduced with either Ad-siBAG3 or Ad-control for 3 days. Transduced NRVCs were then incubated with the translation inhibitor cycloheximide (10 µg/ml) for different time intervals (0, 30 min, 45 min, 1 hour, 2 hours, 4 hours, 6 hours and 8 hours) and the expression of autophagy marker LC3 II and Cx43 proteins were measured by Western blot. LC3 II and Cx43 levels for each condition (Ad-siBAG3 or Ad-control) at each time point were quantified and normalized to their levels at time zero (n = 5). α-tubulin served as a loading control. *p < 0.05; **p < 0.01; ***p < 0.001.

Based on our observations that knock-down of BAG3 results in reduced levels of Cx43, we performed RT-qPCR to evaluate Cx43 mRNA changes as a result of BAG3 suppression. However, suppression of BAG3 did not lead to significant changes in Cx43 mRNA levels (Fig. S2). Co-immunoprecipitation results indicated that BAG3 and Cx43 form a complex together in NRVCs (Fig. 2A). Considering the role of BAG3 as a co-chaperone in protein stabilization^24^, we next investigated whether BAG3 affects the stability of Cx43. To this end, transduced NRVCs were treated with the translation inhibitor, cycloheximide, at different time intervals and Cx43 levels were then evaluated by Western blot. Our data indicate that the levels of Cx43 protein were significantly lower in cardiomyocytes with BAG3 knockeddown compared to control cells, with the exception of the 6 hour post cycloheximide time point. Furthermore, we investigated the effect of BAG3 suppression on LC3 levels and found that LC3 II levels were significantly reduced in siBAG3-transduced NRVCs compared to control cells when cells were treated with cycloheximide (Fig. 2B–E). It has been previously reported that BAG3, by stabilizing mRNA, controls LC3 translation in HeLa cells. Cycloheximide treatment impaired an increase in LC3 levels as a result of BAG3 overexpression^25^. We also evaluated the protein levels of BAG3’s binding partner, HSP70, in control as well as BAG3-suppressed cardiomyocytes and did not detect any significant reduction in HSP70 levels as a result of cycloheximide treatment, either in control or BAG3-suppressed cardiomyocytes (Fig. S3A–C). Taken together, our results suggest that BAG3 plays a key role in controlling the stability of both Cx43 and LC3 II in primary cardiomyocytes.

### Cx43 dynamics under nutrient starvation

**Figure 3:**
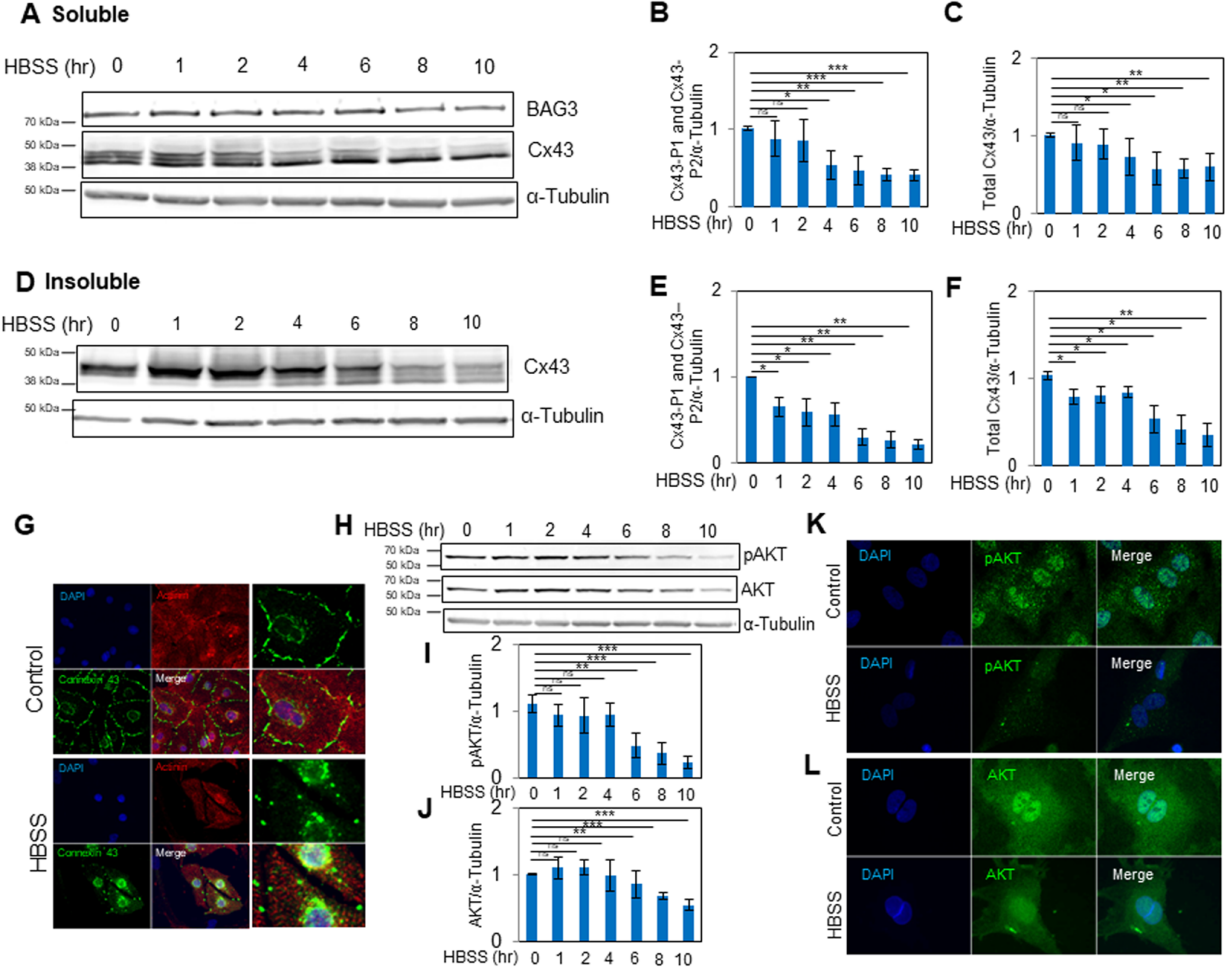
Alteration of Cx43 under conditions of nutrient starvation. NRVCs were starved in HBSS medium at different time intervals (0, 1, 2, 4, 6, 8 and 10 hours). (**A**) Protein levels of Cx43 were measured in soluble fractions of cell lysates by Western blot. The levels of (**B**) Cx43-P1, Cx43-P2 and (**C**) total Cx43 in soluble fractions of cell lysates were quantified based on the data shown in (A) (n = 5). (**D**) Protein levels of Cx43 were measured in insoluble fractions of cell lysates by Western blot. The levels of (**E**) Cx43-P1, Cx43-P2 and (**F**) total Cx43 in insoluble fractions of cell lysates were quantified based on the data shown in (**D**) (n = 3). (**G**) NRVCs were starved for 8 hours in HBSS medium and Cx43 localization was monitored by immunocytochemistry. Microscopic imaging with antibodies against actinin and Cx43 indicated that under starvation, Cx43 levels reduced in plasma membrane while Cx43 localization increased in the perinuclear area of cardiomyocytes. (**H**) Protein levels of pAKT and AKT were measure in soluble fractions of cell lysates by Western blot. The levels of (**I**) pAKT and (**J**) AKT in soluble fractions of cell lysates were quantified (n = 4). NRVCs were starved for 8 hours in HBSS medium and (**K**) pAKT and (**L**) AKT localizations were monitored using immunofluorescence analysis. α-tubulin served as a loading control for both the soluble and insoluble fractions. *p < 0.05; **p < 0.01; ***p < 0.001.

In order to determine the molecular mechanism underlying the turnover of Cx43 under pathological conditions, NRVCs were exposed to HBSS medium as a means of inducing nutrient starvation and Cx43 expression and localization were investigated. Western blot analysis of the soluble cell fraction indicated that starvation led to a reduction in both Cx43-P1 and Cx43-P2, while Cx43-P0 levels increased over time. At 10 hours post starvation, there was a 59% decrease in the level of Cx43-P1 and Cx43-P2 and a 40% decrease in the level of total Cx43. No significant change in the expression of BAG3 was found (Fig. 3A–C). We also analyzed the insoluble cell fraction and found that, consistent with data from the soluble fraction, Cx43-P1 and Cx43-P2 were reduced as a result of starvation (Fig. 3D–F). Immunocytochemistry revealed that Cx43 localized to the plasma membrane and perinuclear area in control cells. In starved cardiomyocytes, the level of membrane-bound Cx43 was reduced and Cx43 aggregates were observed in the perinuclear area (Fig. 3G). Previous studies have reported dephosphorylation^26^ and autophagic degradation^27^ of Cx43 during ischemic stress conditions. However, the precise pathways and key molecular players remain unknown. AKT has been reported to phosphorylate Cx43 on Ser373^28^. We therefore investigated the effect of HBSS starvation on protein levels of AKT and phospho-AKT (pAKT). Western blot and microscopic analyses indicated that the levels of AKT and pAKT were reduced as a result of HBSS starvation (Fig. 3H–L). Our results indicated that although dephosphorylation of Cx43 increased as a result of nutrient starvation for 10 hours, total Cx43 expression also exhibited a 65% reduction suggesting that both degradation and dephosphorylation regulate the fate of Cx43 under stress condition. Taken together, these results indicate that starvation leads to a decrease in total levels of Cx43 as well as alteration in its phosphorylation state.

### BAG3 suppression impairs phosphorylation state and degradation of Cx43 under nutrient starvation

**Figure 4:**
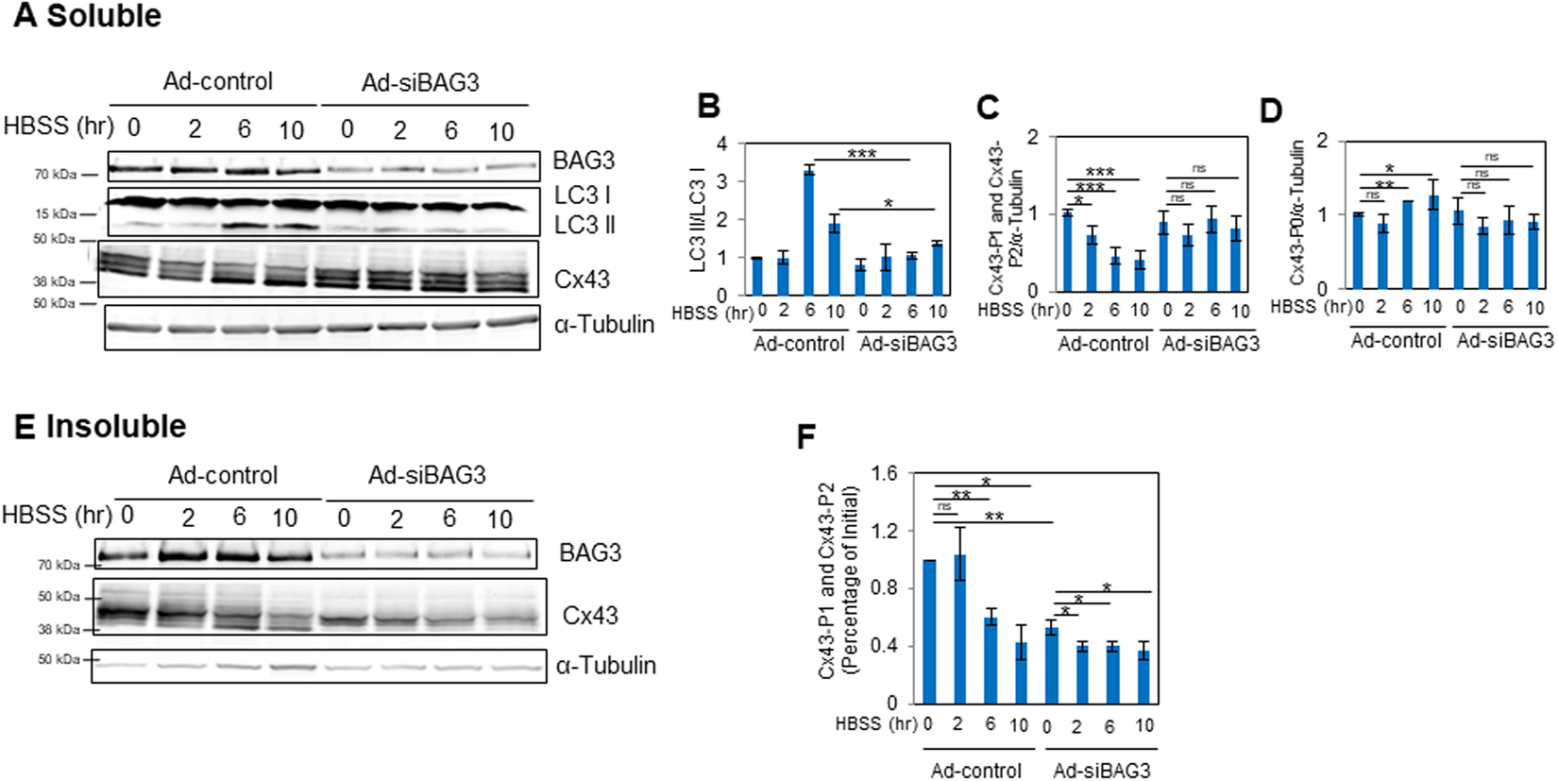
Knock-down of BAG3 impairs Cx43 dynamics under stress conditions. NRVCs were transduced with either Ad-siBAG3 or Ad-control for 3 days and then cells were starved in HBSS medium for 2, 6 or 10 hours. (**A**) The levels of the autophagy markers, LC3 II and LC3 I, Cx43 and BAG3 were measured in soluble fractions of cell lysates by Western blot. (**B**) The levels of LC3 II/LC3 I in soluble fractions of cell lysates were quantified based on the data shown in (A) (n = 3). (**C**) The levels of Cx43-P1 and Cx43-P2 in soluble fractions of cell lysates were quantified based on the data shown in (A) (n = 5). (**D**) The levels of Cx43-P0 in soluble fractions of cell lysates were quantified based on the data shown in (A) (n = 5). (**E**) BAG3 and Cx43 levels in insoluble fractions of cell lysates were measured by Western blot. (**F**) Cx43-P1 and Cx43-P2 levels in insoluble fractions of cell lysates were quantified based on the data shown in (E) (n = 3). α-tubulin served as a loading control for both soluble and insoluble fractions. *p < 0.05; **p < 0.01; ***p < 0.001 (n = 3).

BAG3 has been reported to be a critical regulator of autophagy^14^. In order to determine whether BAG3 plays a role in Cx43 degradation under pathological conditions, NRVCs were transduced with either Ad-control or Ad-siBAG3 for 3 days. Transduced cardiomyocytes were then starved in HBSS medium for different time intervals of 2, 6, or 10 hours and Cx43 degradation was investigated by Western blot. We first evaluated the effect of BAG3 on activation of autophagy by measuring the ratio of LC3 II/LC3 I. LC3 II is the lipidated form of LC3 I and is involved in autophagosome membrane fusion events during autophagy. The LC3 II/LC3I ratio is indicative of autophagic activity and autophagosome formation. Analysis of the soluble fraction revealed that LC3 II/LC3 I ratio was significantly increased in control cardiomyocytes at 6 and 10 hours after starvation, while no such increase was observed in BAG3-suppressed cardiomyocytes (Fig. 4A,B). In addition, Cx43-P1 and Cx43-P2 levels were significantly reduced at 2, 6, and 10 hours after starvation in control cardiomyocytes while no significant reduction in Cx43-P1 and Cx43-P2 levels were observed in Ad-siBAG3-transduced cardiomyocytes. We also investigated the levels of Cx43-P0 as a result of starvation and found that Cx43-P0 was significantly upregulated in control cardiomyocytes while no significant increase in Cx43-P0 was detected in cardiomyocytes suppressed for BAG3 (Fig. 4A–D). In addition, data from the insoluble cell fraction indicate that at 10 hours after starvation, there was a 58% reduction in Cx43-P1 and Cx43-P2 in control cardiomyocytes while there was a 30% reduction in cells with BAG3 knocked down, suggesting that BAG3 suppression interferes with Cx43 degradation under stress conditions (Fig. 4E,F).

### Tubulin depolymerization dysregulates Cx43

**Figure 5:**
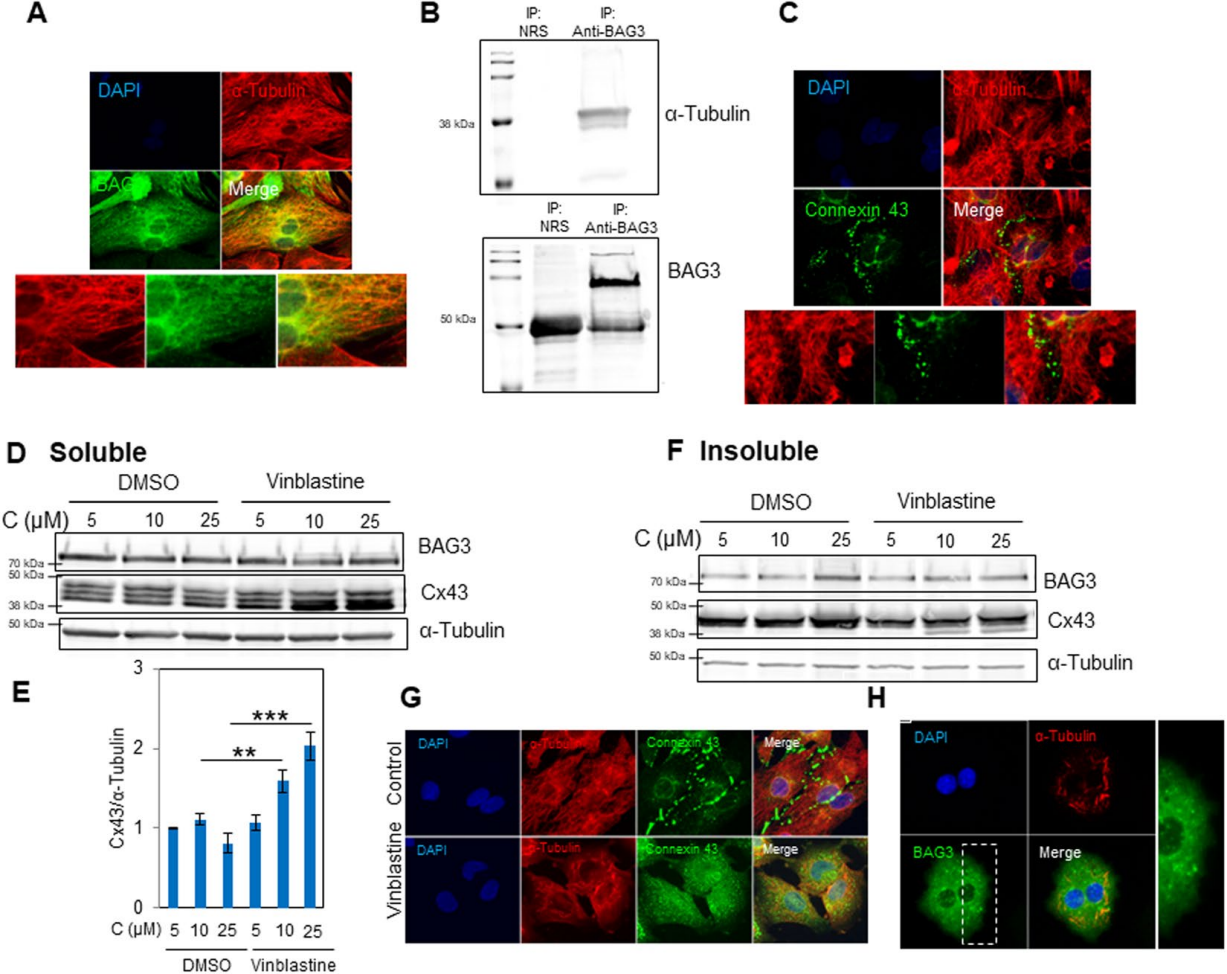
BAG3 colocalizes with the tubulin network and tubulin depolymerization dysregulates Cx43. (**A**) Immunocytochemistry labeling using antibodies against α-Tubulin and BAG3 proteins showed that BAG3 colocalized with α-Tubulin cytoskeleton network. (**B**) Whole cell extracts from NRVCs were immunoprecipitated with an antibody against BAG3 or normal rabbit serum (NRS) followed by Western blot analysis of immunocomplexes using antibodies against BAG3 or α-tubulin. (**C**) Immunocytochemistry labeling by using antibodies against α-Tubulin and Cx43 proteins showed that Cx43 colocalized with α-Tubulin in the perinuclear area. NRVCs were treated for 12 hours with 5, 10 and 25 µM microtubule depolymerizing agent, vinblastine. (**D**) BAG3 and Cx43 levels were measured in soluble fractions of cell lysates by Western blot. (**E**) Total Cx43 protein levels were quantified in soluble fractions of cell lysates based on the data shown in (D) (n = 3). (**F**) BAG3 and Cx43 levels were measured in insoluble fractions of cell lysates. α-tubulin served as a loading control for both soluble and insoluble fractions. (**G**) Immunocytochemistry using antibodies against α-Tubulin and Cx43 proteins showed that Cx43 accumulated in cellular cytoplasm as a result of vinblastine treatment. (**H**) Immunocytochemistry using antibodies against α-Tubulin and BAG3 showed that BAG3 aggregates accumulated in cellular cytoplasm as a result of vinblastine treatment. **p < 0.01; ***p < 0.001.

BAG3 transports protein aggregates to lysosomes for removal through the autophagy pathway by interacting with microtubules^29^. Next, we performed a series of experiments to explore the importance of tubulin in regulating Cx43 turnover. Immunocytochemistry using antibodies against BAG3 and α-Tubulin indicated that BAG3 colocalized with the tubulin network in primary cardiomyocytes (Fig. 5A). Co-immunoprecipitation using an antibody to pull down the BAG3 protein indicated that BAG3 interacts with the tubulin network in NRVCs (Fig. 5B). We also investigated the localization of Cx43 and tubulin and found that Cx43 was surrounded by the tubulin network in the perinuclear area (Fig. 5C). We further investigated the impact of tubulin on Cx43 protein homeostasis by impairing the assembly of microtubules using vinblastine. NRVCs were treated with 5, 10, or 25 µM vinblastine for 12 hours and levels of Cx43 were determined in both the soluble and insoluble cell fractions. Data indicated that the levels of Cx43 significantly increased in response to vinblastine at 10 and 25 µM concentrations. In addition, we found that Cx43-P0 levels were upregulated in the insoluble fractions as a result of vinblastine treatment (Fig. 5D–F). Immunofluorescence analysis indicated that Cx43 localization to the plasma membrane was inhibited and Cx43 accumulated in the cytoplasm as a result of microtubule depolymerization (Fig. 5G). In addition, the accumulation of BAG3 aggregates was observed in vinblastine-treated cardiomyocytes (Fig. 5H). These data suggest that BAG3 and tubulin are two important regulators of Cx43 in cardiomyocytes.

### BAG3 suppression impairs gap junction function

**Figure 6:**
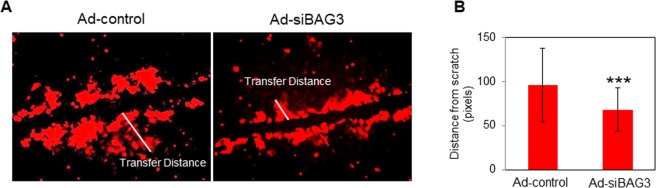
Knock down of BAG3 impairs gap junction-mediated dye transfer. (**A**) NRVCs were transduced with either Ad-siBAG3 or Ad-control. 3 days post transduction, confluent cardiomyocytes were scrape loaded with sulforhodamine B dye followed by imaging using fluorescent microscope. (**B**) Image J quantification indicated that dye transfer distance was significantly less in BAG3-suppressed cardiomyocytes compared to that in control cells. ***p < 0.001 (n = 56).

To determine whether BAG3 suppression impacts the function of gap junctions in cardiomyocytes, we measured transfer distance of the low molecular weight dye, sulforhodamine B, by using a scrape-loading technique^30^. For this purpose, scratches were made in a confluent culture of transduced cardiomyocytes using a surgical scalpel. NRVCs were then incubated with sulforhodamine B dye followed by imaging. Image J analysis indicated that the dye transfer distance was significantly reduced as a result of BAG3 suppression; suggesting that BAG3 may impact intercellular communication of cardiomyocytes by impairing the quality of the gap junction protein, Cx43 (Fig. 6A,B).

## Discussion

Gap junctions propagate action potentials through the myocardium and maintain cardiac rhythm by connecting the plasma membrane of cardiomyocytes^2^. Cx43 is highly expressed in cardiomyocytes and has a very dynamic turnover with a short half-life^4^. Therefore, exploring the key molecular players that regulate turnover of Cx43 is of great importance for understanding proper heart function. In this study, we have identified the importance of BAG3 in regulating turnover of Cx43 under normal, as well as stress conditions, in primary cultures of neonatal cardiomyocytes using several complementary approaches. BAG3 interacts with the HSP70/HSC70 chaperone complex through its BAG domain^22^. Mutations in BAG3 dysregulate the function of the HSP70/HSC70 chaperone complex and impair protein homeostasis leading to the accumulation of protein aggregates and development of cardiomyopathy^24^. Our data indicate that Cx43 levels were downregulated as a result of BAG3 knock-down. By inhibiting translation, we found that the stability of Cx43 was significantly reduced in BAG3-suppressed cardiomyocytes compared to control cells. Whether BAG3 directly impacts Cx43 stability or the absence of BAG3 destabilizes Cx43 by impairing the chaperone activity of the HSP70/HSC70 complex remains in question. Interestingly, a recent study showed that deletion of a member of the heat shock protein family, HSPB7, resulted in Cx43 reduction in mice followed by impairment of cardiac electrical conduction and development of heart failure^31^.

The role of BAG3 in PQC in cardiomyocytes has been previously reported^13^. However, to our knowledge, there is no report describing the role of BAG3 in gap junction quality control. Recent studies have shown that BAG3 impacts autophagosome formation and autophagic turnover by regulating LC3 lipidation through a translational mechanism^25^. We found that when lysosomal activity was inhibited, the level of Cx43 was significantly increased and Cx43 aggregates accumulated in the cytoplasm. Furthermore, immunocytochemistry demonstrated co-localization of Cx43 aggregates with ubiquitin in the perinuclear area. Ubiquitination of Cx43 has been demonstrated to play an important role in Cx43 internalization^32^; however other studies have reported that ubiquitination of Cx43 is not critical for the regulation of its turnover^33^. When BAG3 was knocked down using adenovirus transduction of BAG3 siRNA, lysosomal turnover of Cx43 was inhibited. Considering the rapid turnover of Cx43 in heart, impairment of Cx43 turnover adversely affected cell-to-cell communication in cardiac tissue and contributed to arrhythmia and heart failure^3^. Further experiments by inhibiting proteasomal activity suggested that degradation of Cx43 in neonatal cardiomyocytes could also occur through the proteasome, as it has been reported previously^5^. However, previous studies have reported that proteasome inhibitors may interfere with internalization of gap junction proteins and subsequent degradation^34^; therefore, whether Cx43 is a direct target for proteasomal degradation in NRVCs remains to be further investigated.

When cardiomyocytes were subjected to starvation, Cx43 became dephosphorylated and total Cx43 levels was reduced as well. Immunocytochemistry indicated that Cx43 localization in the perinuclear area increased as a result of starvation. It was previously reported that Cx43 degrades through the autophagy pathway during ischemia^27^. BAG3 plays a protective role under cardiac stress conditions by activating autophagy^35^. Therefore, we investigated the effect of BAG3-mediated autophagy on the fate of Cx43 under stress conditions. Western blot data of the levels of autophagy markers, LC3 I and LC3 II, indicated that autophagosome formation was significantly higher in control cells compared to cardiomyocytes with BAG3-knocked down. We then found that Cx43 degradation was significantly impaired in BAG3-suppressed cardiomyocytes. In addition, our data indicate that knock down of BAG3 significantly impaired Cx43 dephosphorylation. During ischemia, protein phosphatases interact with Cx43 and cause its dephosphorylation^36^. However, further experiments are required to explore the effect of BAG3 on the activity of phosphatases and the phosphorylation state of Cx43.

Microtubules interact directly with Cx43 and control its trafficking and translocation into gap junctions^37^. In addition, it has been reported that BAG3 interacts with the microtubule motor, dynein, and delivers misfolded proteins into aggresomes for further degradation and elimination by autophagy. Aggresome formation was impaired when microtubules were depolymerized^29^. We found that BAG3 colocalized with α-Tubulin in NRVCs and tubulin disassembly resulted in alteration of Cx43. The accumulation of non-phosphorylated Cx43 within gap junctions is associated with arrhythmogenesis and heart failure^26^. These data suggest that BAG3 and tubulin are important regulators of Cx43 in primary cardiomyocytes.

**Figure 7:**
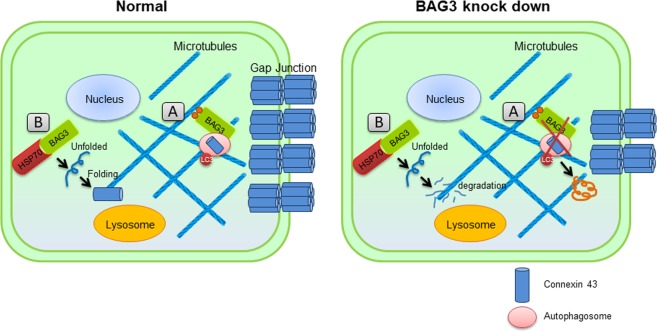
Schematic model representing the role of BAG3 in quality control and stability of Cx43 protein. (**A**) BAG3, through association with microtubules, delivers misfolded and damaged proteins for further degradation and removal through autophagy-lysosome pathway. Impaired autophagic degradation of proteins in the absence of BAG3 resulted in the accumulation of insoluble aggregates. (**B**) BAG3 functions as a co-chaperone of HSP70/HSC70 chaperone complex and plays an important role in protein stabilization. BAG3 suppression results in the destabilization of Cx43; leading to Cx43 degradation and reduction in Cx43 level.

In summary, we report the impact of BAG3 on regulating the stability of the gap junction protein, Cx43. Considering the dynamic structure of gap junctions, their quality control is of great importance in cardiac synchronous contraction. We found that there was a significant reduction in Cx43 levels and lysosomal turnover in cardiomyocytes with reduced levels of BAG3. In addition, the stability of Cx43 was significantly reduced in BAG3-suppressed cardiomyocytes (Fig. 7). Taken together, our findings suggest that BAG3 plays an important role in modulating the fate of Cx43, and enhancing BAG3 levels under stress conditions and might be a promising target to improve heart function.

## Materials and Methods

### Isolation of cardiomyocytes and culture conditions

NRVCs were isolated from 1–2 day old Sprague-Dawley rats (Charles River) following the protocol described previously^38^. Isolated cardiomyocytes were cultured in Dulbecco’s modified Eagle’s medium (DMEM, Life Technologies, Carlsbad, CA) supplemented with 2% fetal bovine serum (FBS, Denville Scientific Inc., Hollistion, MA) and 25 µg/ml gentamicin (Life Technologies).

### Adenoviral transduction

NRVCs were transduced with Ad-siBAG3 (Vector Biolabs, Malvern, PA) in reduced volumes of FBS-free DMEM at 37 °C for 2 hours. The medium was then replaced with DMEM with 2% FBS and 25 µg/ml gentamicin. As an internal control, cells were transduced with Ad-GFP (Vector Biolabs, Malvern, PA). Transduced cardiomyocytes were subjected to other treatments 3 or 5 days post transduction.

### Western blot

Protein extraction from cardiomyocytes was performed as described previously^39^. NRVCs were washed with cold 1X PBS and lysed in RIPA buffer (25 mM Tris-HCl, 150 mM NaCl, 1% NP-40, 1% sodium deoxycholate, 0.1% SDS) supplemented with mammalian protease inhibitor cocktail (Sigma-Aldrich). After 30 min, cells were rotated at 4 °C, spun down at 14000 rpm for 10 min and supernatants were kept as soluble fractions of cell lysates. In order to prepare insoluble fractions, lysis buffer insoluble pellets were washed with cold 1X PBS and dissolved in 2% (w/v) SDS for 30 min at room temperature (RT). Protein concentrations were determined using Bio-Rad Protein Assay Dye. Equal amounts of protein lysates were separated using 10% and 12% SDS polyacrylamide gels and transferred onto wet nitrocellulose membranes (LI-COR) using Bio-Rad’s Western blot system. Membranes were incubated for 3 hours with primary antibody (diluted 1:1000) and 1 hour with secondary antibody (diluted 1:10000) followed by washing with PBST containing 0.5% Tween 20 (1x, 5 min) and 1X PBS (3x, 5 min ea.) after each antibody incubation. The following antibodies were used: BAG3 (Proteintech, 10599-1-AP), LC3 (Sigma, L8918), Cx43 (Abcam, ab11370), α-Tubulin (Sigma, T6074), HSP70 (Santa Cruz, sc-24), Actinin (Sigma, A7732), AKT (Cell Signaling, 9272), pAKT (Cell signaling, 9271), N-cadherin (Abcam, ab76057) and Phospho-Connexin 43 (Ser368, Cell Signaling, 3511).

### Immunocytochemistry

NRVCs were grown in 2-well chamber slides (Lab-Tek^®^), fixed with 4% paraformaldehyde (10 min, RT) and permeabilized with 0.5% Triton-X 100 (10 min, RT). Cells were then incubated with 0.1 M glycine (pH 3.5, 30 min, RT), blocked with 1% (w/v) bovine serum albumin solution containing 0.1% Tween 20 (30 min, RT) and labeled for 3 hours at RT with primary antibody (diluted 1:100) and then 1 hour at RT with Alexa Fluor^®^ secondary antibody (diluted 1:200, Thermo Fisher Scientific, Eugene, OR). Cardiomyocytes were then mounted with DAPI-containing VECTASHIELD hard set (Vector Laboratories) and imaged using a Leica fluorescent microscope (Leica Microsystems, Deerfield, IL). The following antibodies were used: Cx43 (Abcam, ab11370), Actinin (Sigma, A7732), AKT (Cell Signaling, 9272), pAKT (Cell signaling, 9271), BAG3 (Proteintech, 10599-1-AP), Ubiquitin (Santa Cruz, sc-8017), Phospho-Connexin 43 (Ser368, Cell Signaling, 3511) and α-tubulin (Sigma, T6074).

### Co-Immunoprecipitation (Co-IP)

NRVCs were lysed in lysis buffer (20 mM Tris HCl pH 8, 137 mM NaCl, 10% glycerol, 1% Nonidet P-40 and 2 mM EDTA) and rotated with a specific antibody or normal rabbit serum (NRS) as control for 4 hours at 4 °C. The resultant lysates were then rotated with protein A/G beads (Pierce) for 4 hours at 4 °C followed by washing and analysis by western blotting.

### Quantitative reverse transcription-PCR (RT-qPCR)

cDNA was synthesized using 1 µg of isolated RNA and M-MLV Reverse Transcriptase (Invitrogen). RT-qPCR gene expression analysis was then performed using SYBR®Green master mix (Roche) and primers. β-actin served as reference gene. The following primers were used: Cx43 FW: 5′-TCCTTGGTGTCTCTCGCTTT-3′; Cx43 RV: 5′-GTGAGGAGCAGCCATTGAAG-3′.

### Scrape-loading dye transfer

NRVCs were seeded in Lab-Tek^®^ chamber slides and transduced with either Ad-control or Ad-siBAG3 for 3 days. Confluent cardiomyocytes were washed twice with Hank’s Balanced Salt Solution (HBSS, Thermo Fisher Scientific) medium and then scrape-loaded with 1.5 mg/mL sulforhodamine B (MP Biomedicals) followed by incubation at room temperature for 3 min. Cells were rinsed and fixed with PFA 4% at 4 °C and imaged using Keyence BZ-X710 fluorescent microscope. Maximum dye transfer distance was quantified by taking two points in each image using ImageJ analysis.

### Institutional compliance

All experiments were performed in accordance with the guidelines and regulations of the Temple University Institutional Animal Care and Use Committee. All studies have been approved by the Temple University Institutional Animal Care and Use Committee.

### Statistical analysis

Data are presented as mean ± SD. Student’s *t*-test was utilized to calculate the significance of the differences between two groups. *P* value less than 0.05 was considered as statistically significant.

## Supplementary information

Supplementary Material
