# Supplementary material for: Role of Bcl2-associated Athanogene 3 in Turnover of Gap Junction Protein, Connexin 43, in Neonatal Cardiomyocytes
Source: Sci Rep. 2019 May 21;9:7658. doi: 10.1038/s41598-019-44139-w (PMC6529437; doi:10.1038/s41598-019-44139-w)
Supplement: Supplementary file 1 — Supplementary Material [file 41598_2019_44139_MOESM1_ESM.pdf]

Role of Bcl2-associated Athanogene 3 in Turnover of Gap Junction Protein, Connexin 43, in Neonatal Cardiomyocytes

Supplemental Information

Farzaneh G. Tahrir[1], Manish Gupta[1], Valerie Myers[2,3], Jennifer Gordon[1], Joseph Y. Cheung[2,4], Arthur M. Feldman[2,3], and Kamel Khalili[1†]

1. Department of Neuroscience
Center for Neurovirology
Lewis Katz School of Medicine at Temple University
Philadelphia, Pennsylvania

2. Department of Medicine
Lewis Katz School of Medicine at Temple University
Philadelphia, Pennsylvania

3. Cardiovascular Research Center
Lewis Katz School of Medicine at Temple University
Philadelphia, Pennsylvania

4. Center for Translational Medicine
Lewis Katz School of Medicine at Temple University
Philadelphia, Pennsylvania

†Corresponding Author

Mailing address: Department of Neuroscience, Katz School of Medicine at Temple University,

3500 North Broad Street, 7th Floor, Philadelphia, PA 19140 USA

Phone:215.707.4500, Fax: 215.707.4888

Email: kamel.khalili@temple.edu

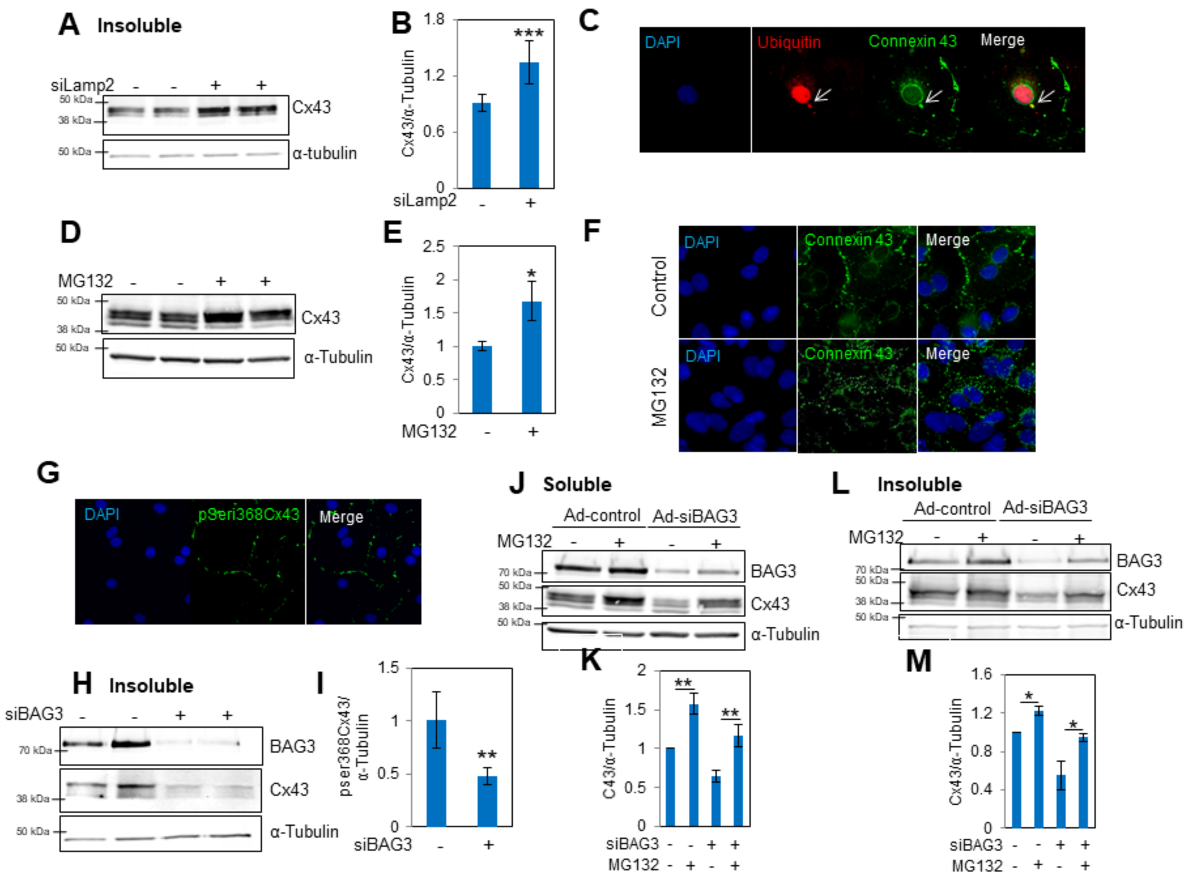

FIGURE S1. Cx43 turnover in neonatal cardiomyocytes. (A) NRVCs were suppressed for lysosomal marker, Lamp2, by using Lamp2 siRNA and Cx43 levels were evaluated in insoluble fractions of cell lysates using Western blot. **(B)** Cx43 levels were quantified based on the data shown in (A) (n=8). **(C) Immunocytochemistry with antibodies for Cx43 and ubiquitin demonstrate colocalization of ubiquitin and Cx43 in aggregates within the perinuclear area. (D)** NRVCs were treated for 12 hours with 5 μM proteasome inhibitor, MG132, and the levels of Cx43 were measured by Western blot. **(E)** Cx43 levels were quantified based on the data shown in (A) (n=4). **(F)** Immunocytochemistry with antibodies for Cx43 indicated that MG132 treatment led to accumulation of Cx43 aggregates inside cardiomyocytes. **(G)** Immunocytochemistry indicated that pSer368Cx43 is highly expressed in the plasma membrane of NRVCs. **(H)** NRVCs were transduced with either Ad-control or Ad-siBAG3 for 3 days and the levels of pSer368Cx43 were evaluated in insoluble protein fractions by Western blot analysis. **(I)**

The levels of pSer368Cx43 were quantified based on the data shown in (H) (n=4). **(J)** NRVCs were transduced with either Ad-control or Ad-siBAG3 for 3 days then treated with MG132 (5 μM, 12 hours). The levels of BAG3 and Cx43 were measured in soluble fractions of cell lysates by Western blot. **(K)** Cx43 levels were quantified based on the data shown in (J) (n=4). **(L)** BAG3 and Cx43 levels were measured in insoluble fractions of cell lysates by Western blot. **(M)** Cx43 levvels were quantified based on the data shown in (L) (n=4). α-tubulin served as a loading control. *p<0.05; **p<0.01; ***p<0.001.

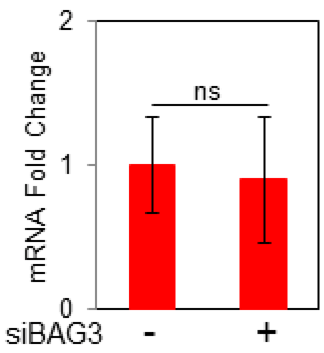

Figure S2. BAG3 knock-down did not alter Cx43 mRNA levels. Cells were transduced with either Ad-control or Ad-siBAG3, and RT-qPCR was performed to investigate Cx43 mRNA changes as a result of BAG suppression (n=9).

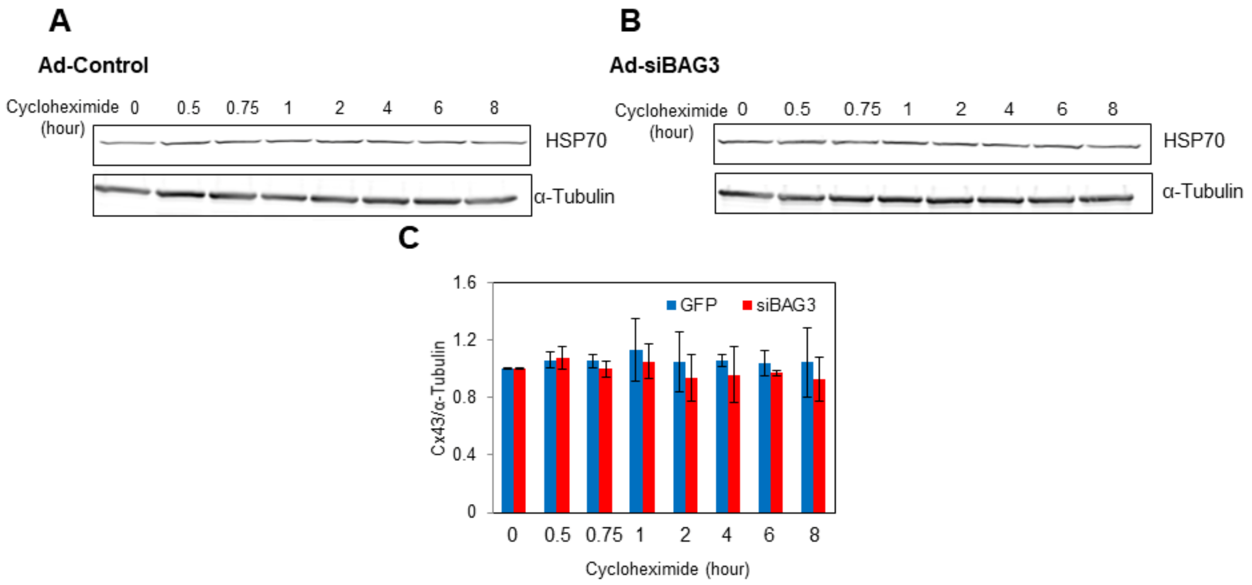

FIGURE S3. Stability of HSP70. (A, B) NRVCs were transduced with either Ad-siBAG3 or Ad-control for 3 days. Transduced NRVCs were then incubated with the mRNA translation inhibitor cycloheximide (10 μg/mL) for different time intervals (0, 30 min, 45 min, 1 hour, 2 hours, 4 hours, 6 hours and 8 hours). The levels of HSP70 in each condition (Ad-siBAG3 or Ad-control) were measured by Western blot. **(C)** HSP70 levels for each condition (Ad-siBAG3 or Ad-control) at each time point were quantified and normalized to their levels at time zero. α-tubulin served as a loading control.

Data for Figures

Figure 1

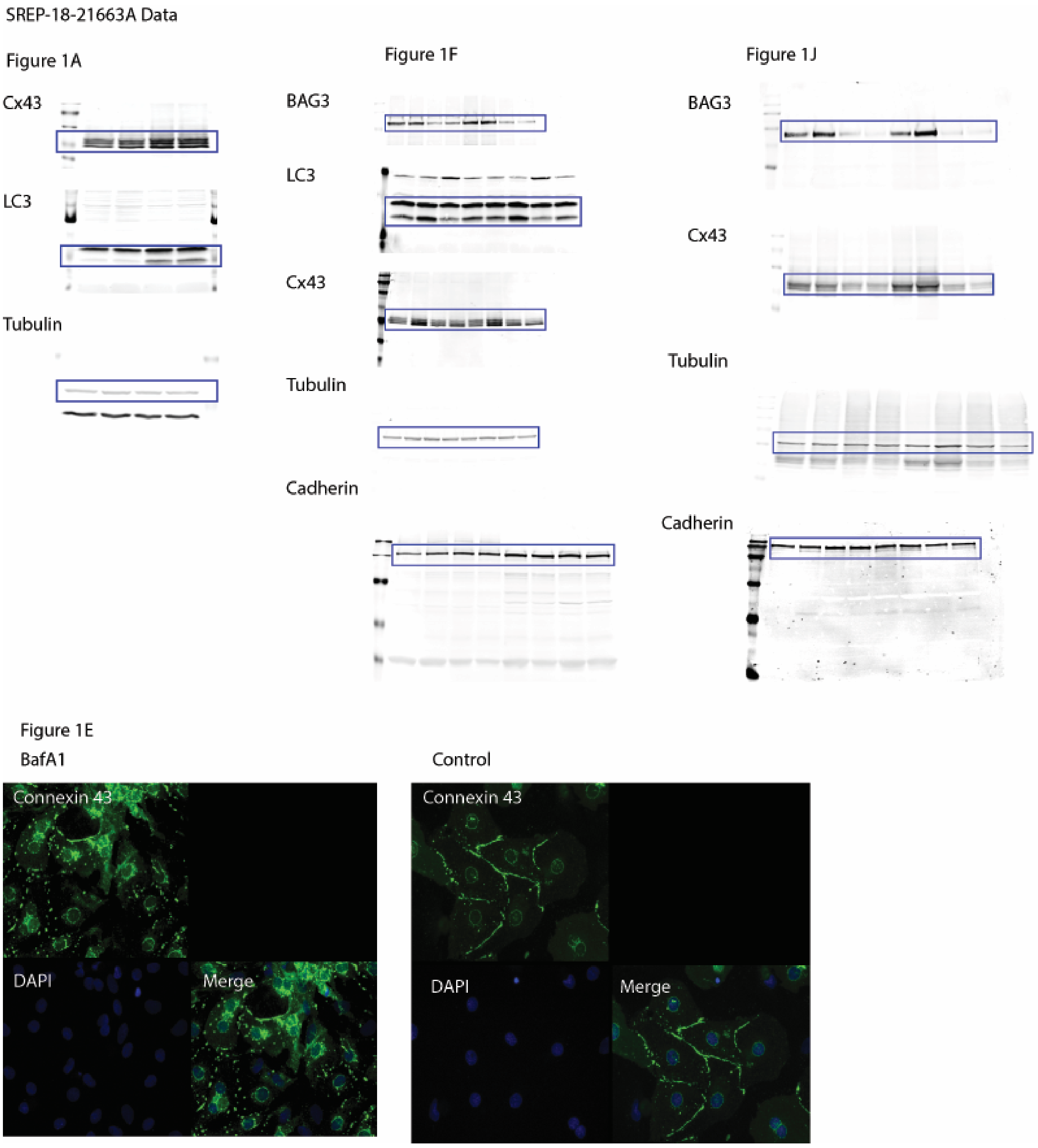
SREP-18-21663A Data
Figure 1A
Cx43
LC3
Tubulin
Figure 1F
BAG3
LC3
Cx43
Tubulin
Cadherin
Figure 1J
BAG3
Cx43
Tubulin
Cadherin
Figure 1E
BafA1
Control
Connexin 43
DAPI
Merge
Connexin 43
DAPI
Merge

Figure 2

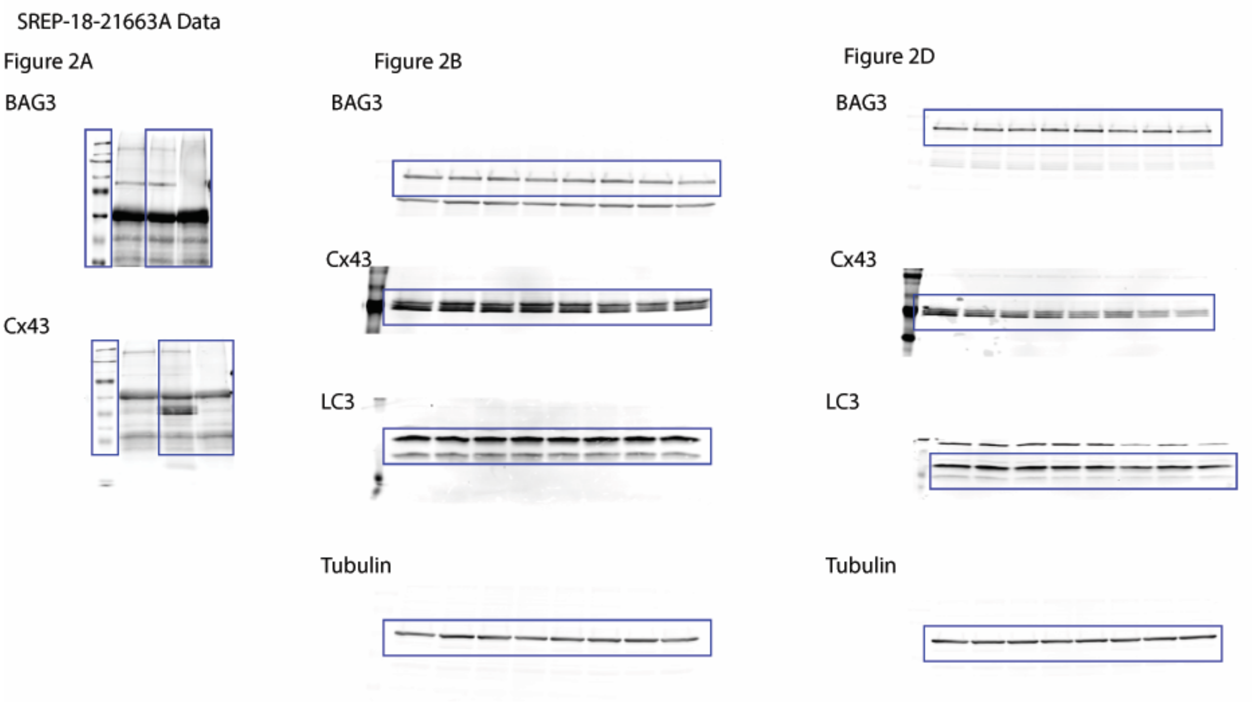

SREP-18-21663A Data
Figure 2A
BAG3
Cx43
Figure 2B
BAG3
Cx43
LC3
Tubulin
Figure 2D
BAG3
Cx43
LC3
Tubulin

Figure 3

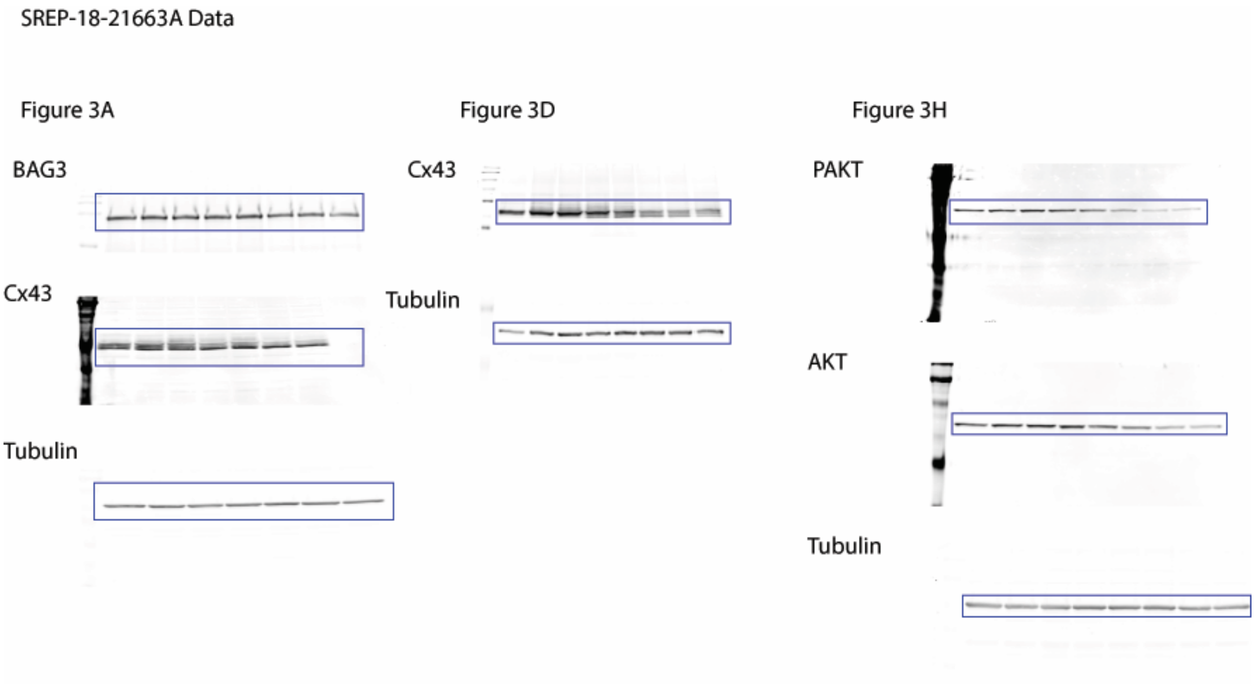
SREP-18-21663A Data
Figure 3A
Figure 3D
Figure 3H
BAG3
Cx43
PAKT
Cx43
Tubulin
AKT
Tubulin
Tubulin

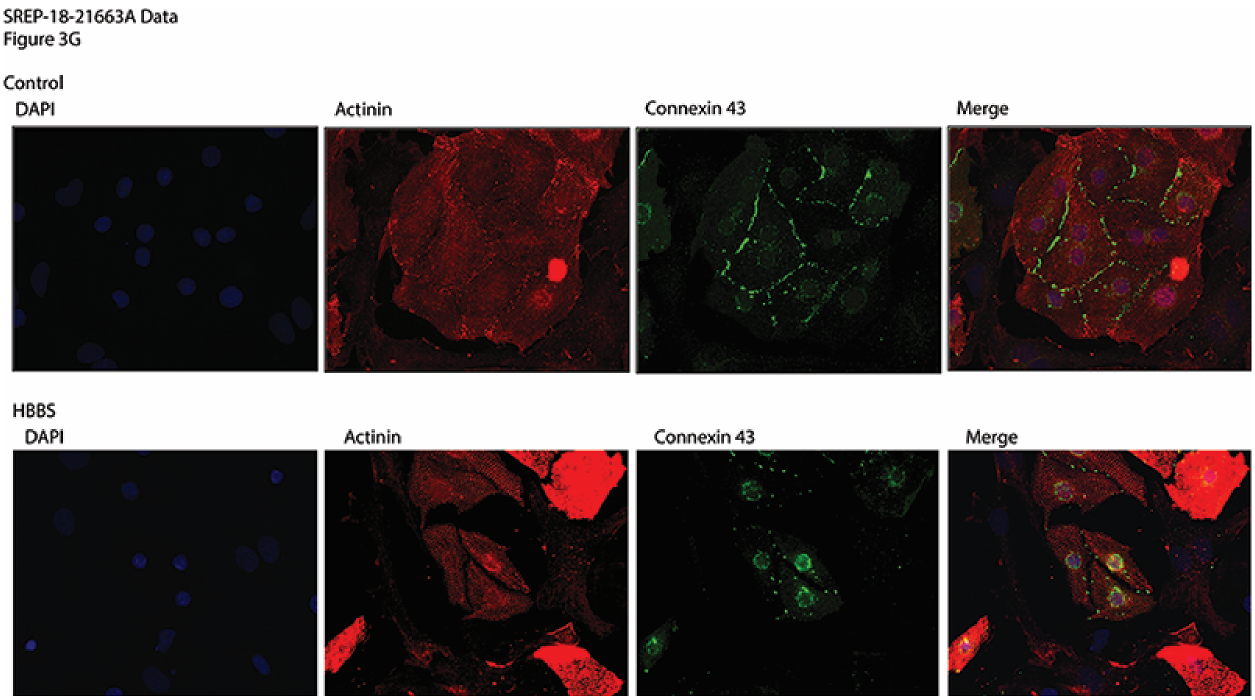
SREP-18-21663A Data
Figure 3G
Control
DAPI
Actinin
Connexin 43
Merge
HBBS
DAPI
Actinin
Connexin 43
Merge

SREP-18-21663A Data
Figure 3K

Control

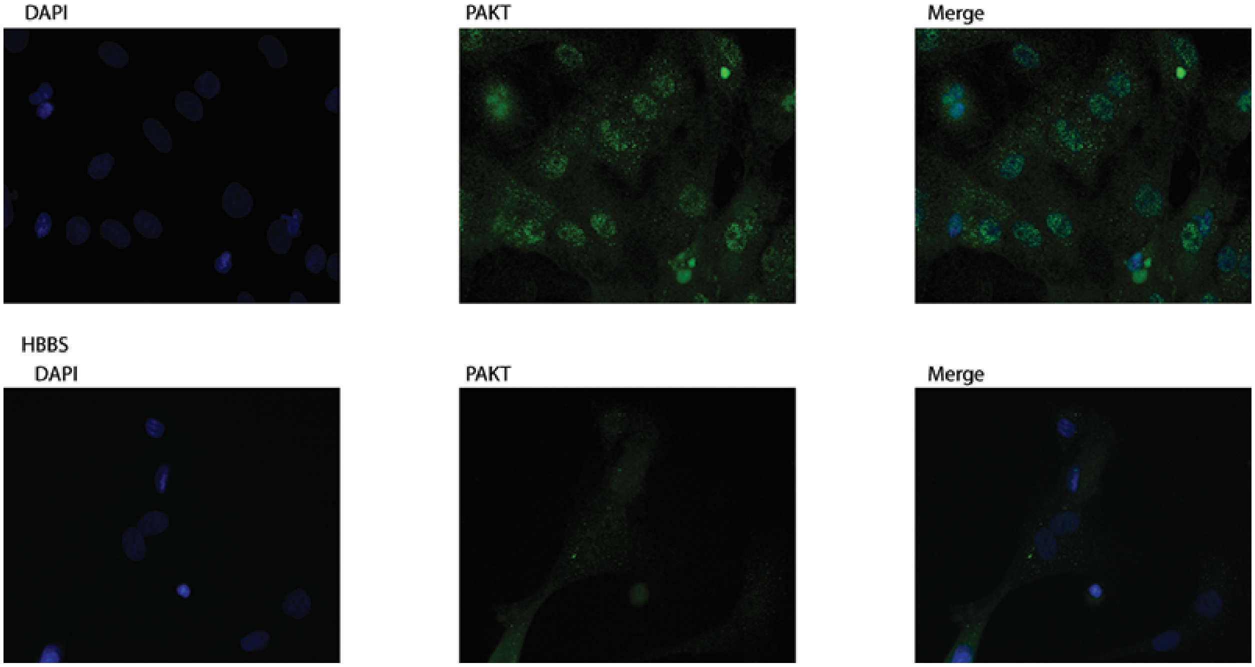

SREP-18-21663A Data
Figure 3L

Control

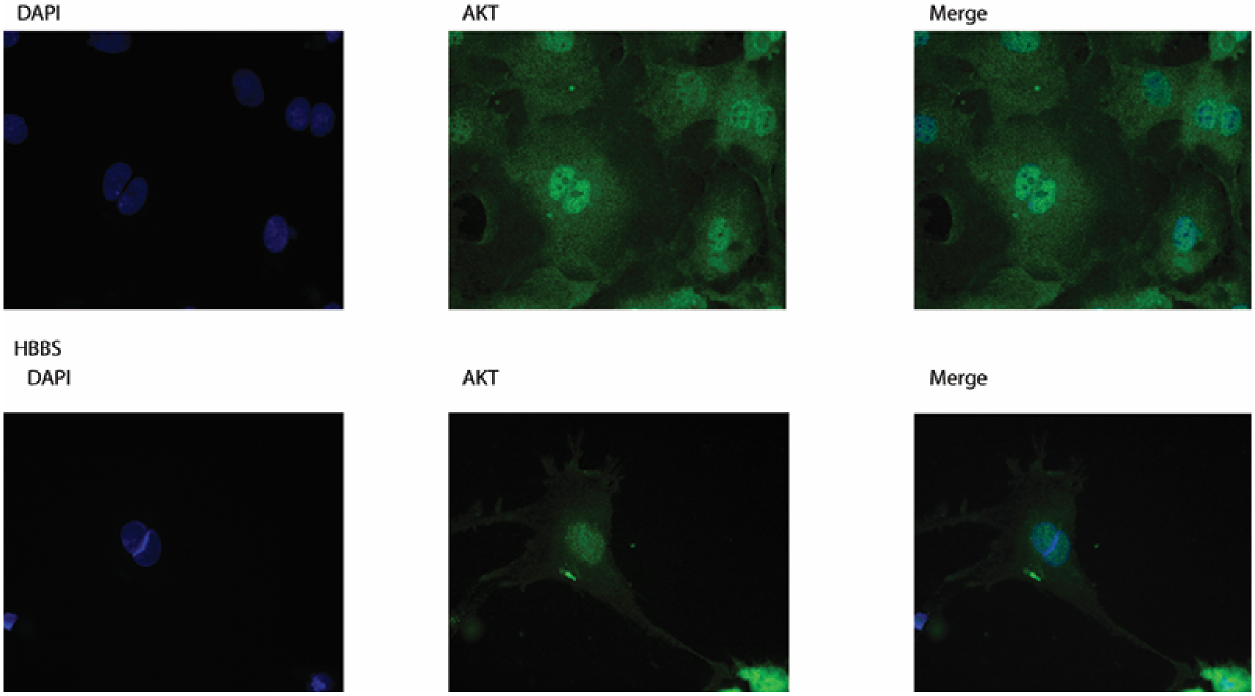

Figure 4

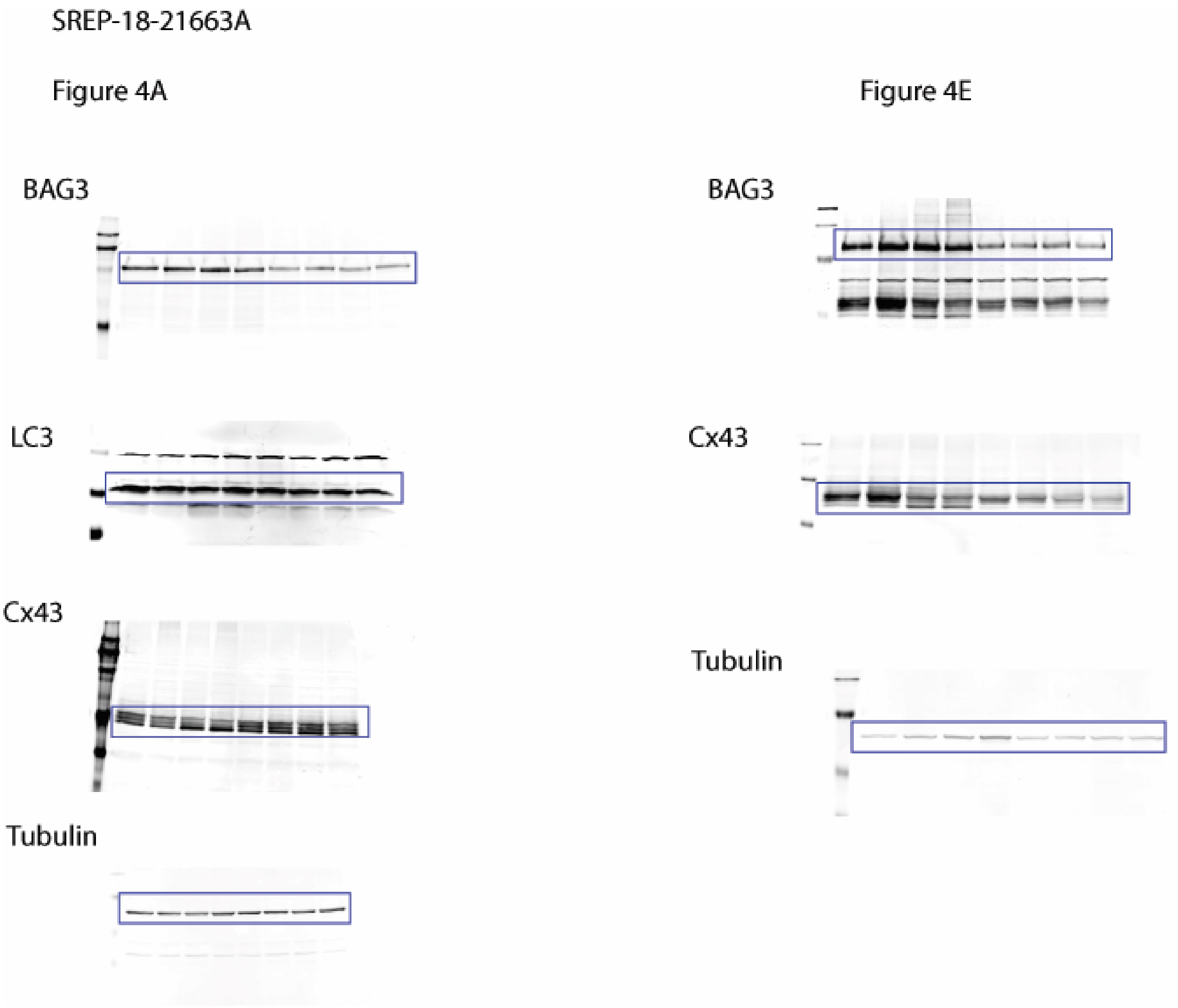
SREP-18-21663A
Figure 4A
Figure 4E
BAG3
BAG3
LC3
Cx43
Cx43
Tubulin
Tubulin

Figure 5

SREP-18-21663A Data
Figure 5A

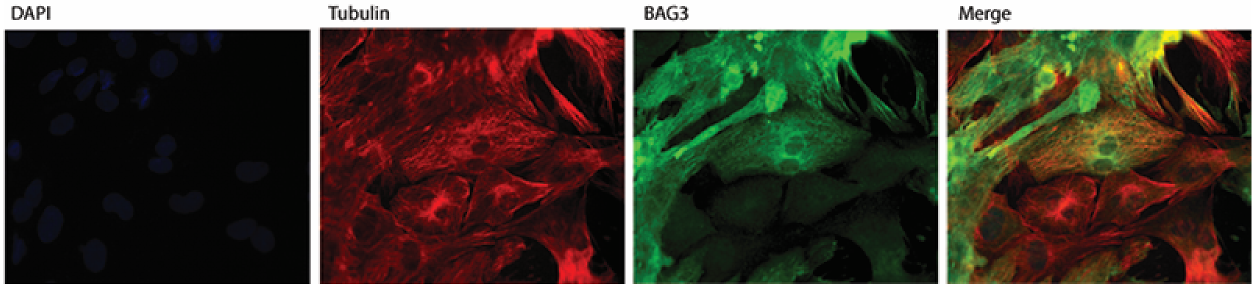

SREP-18-21663A

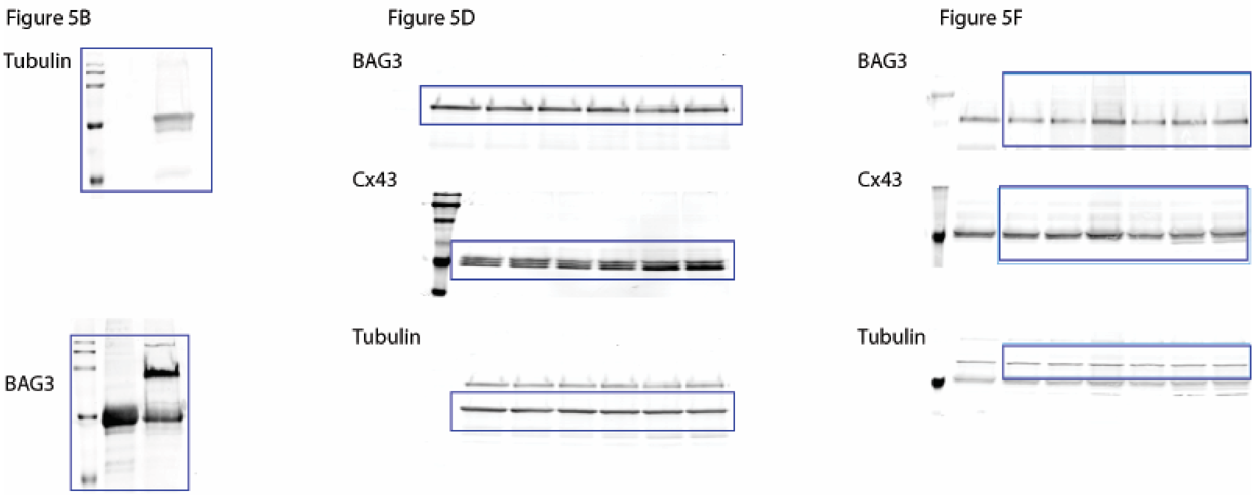

SREP-18-21663A

Figure 5C

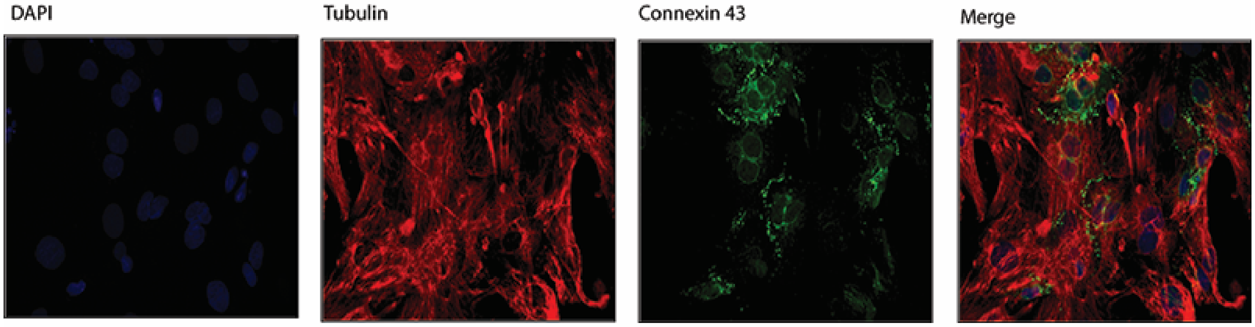

SREP-18-21663A

Figure 5G

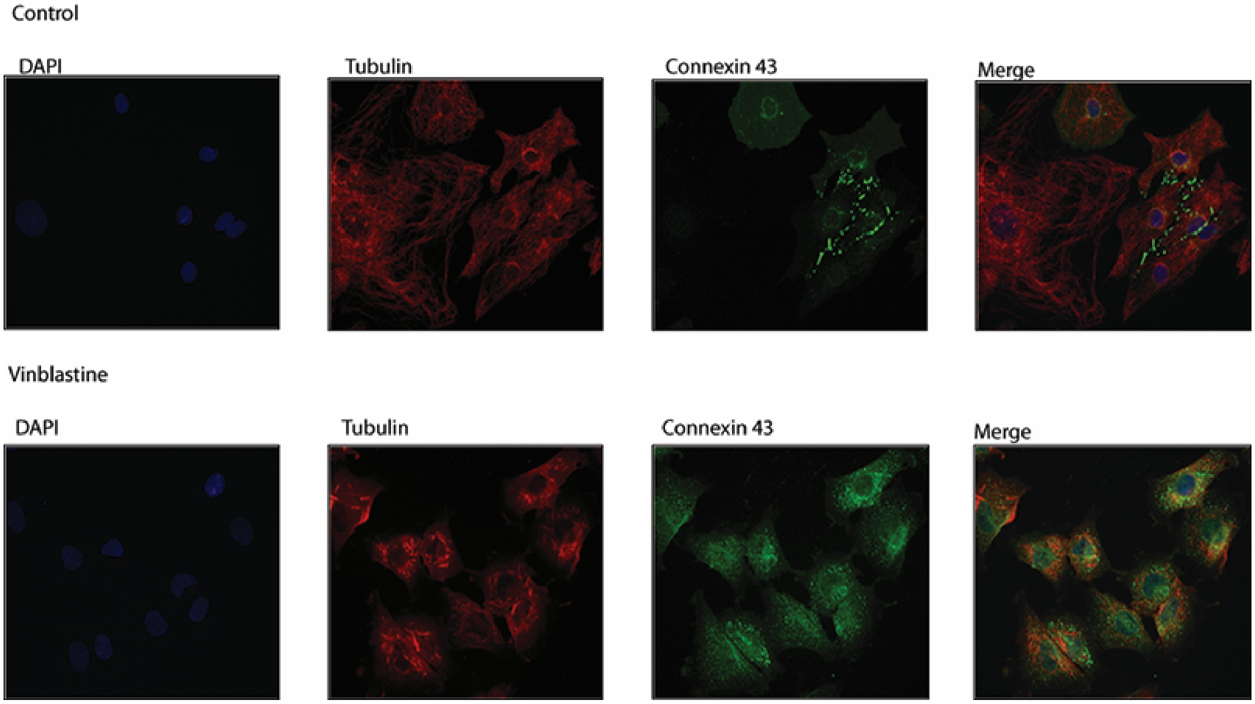

SREP-18-21663A

Figure 5H

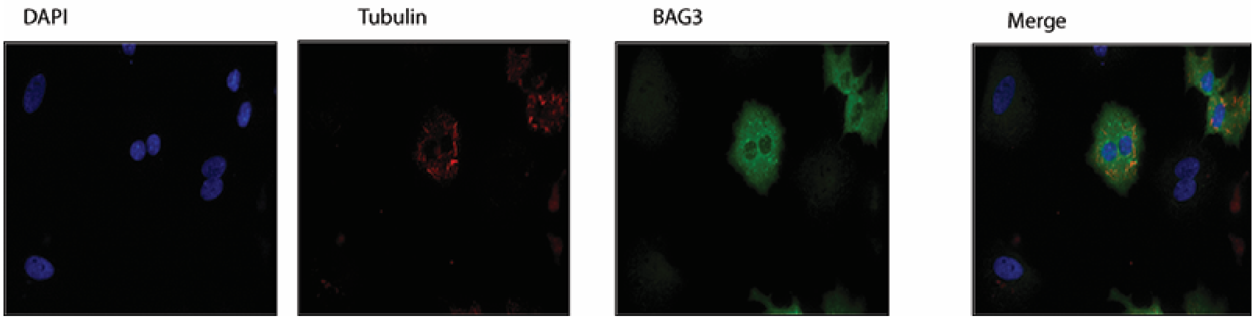

Figure S1

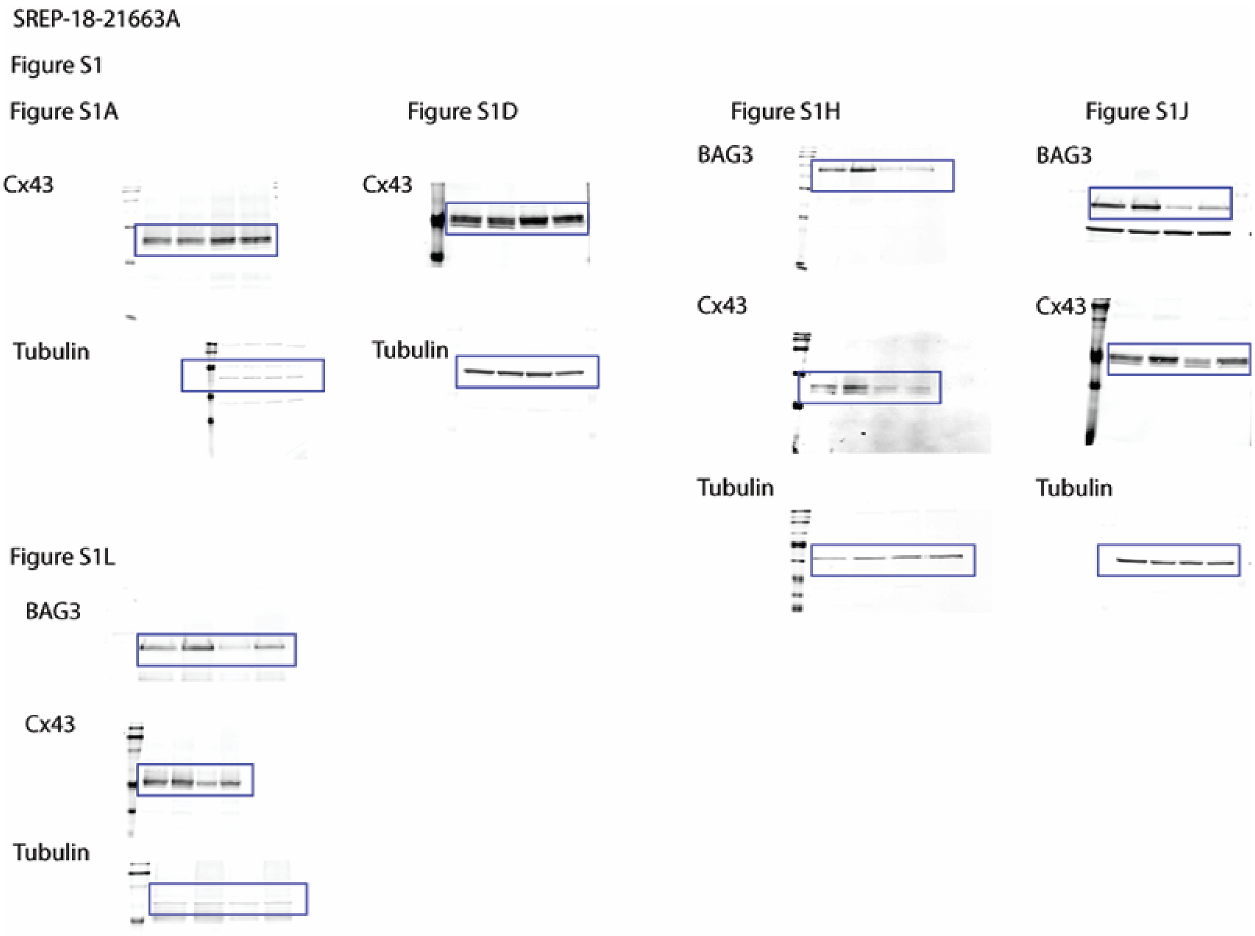
SREP-18-21663A
Figure S1
Figure S1A
Cx43
Tubulin
Figure S1D
Cx43
Tubulin
Figure S1H
BAG3
Cx43
Tubulin
Figure S1J
BAG3
Cx43
Tubulin
Figure S1L
BAG3
Cx43
Tubulin

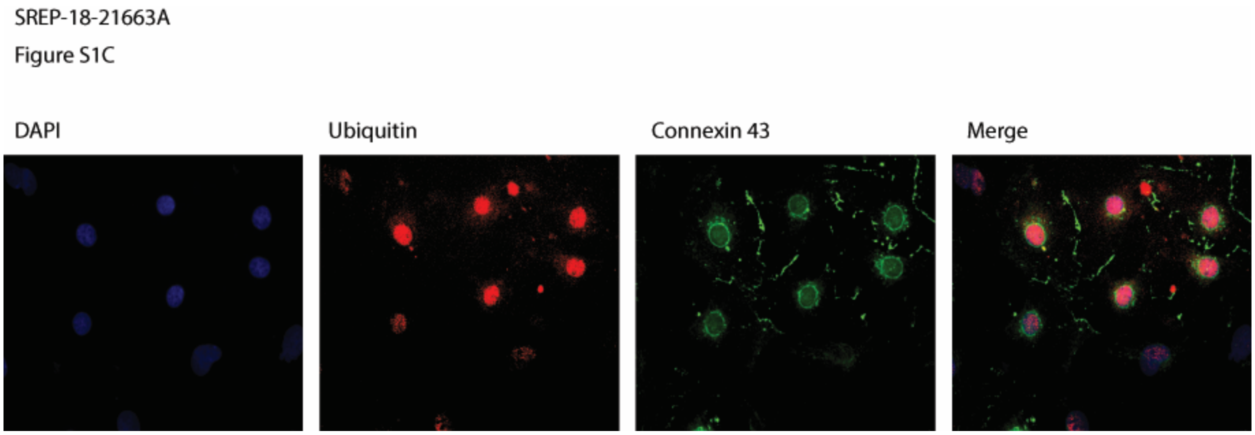
SREP-18-21663A
Figure S1C
DAPI
Ubiquitin
Connexin 43
Merge

SREP-18-21663A

Figure S1F

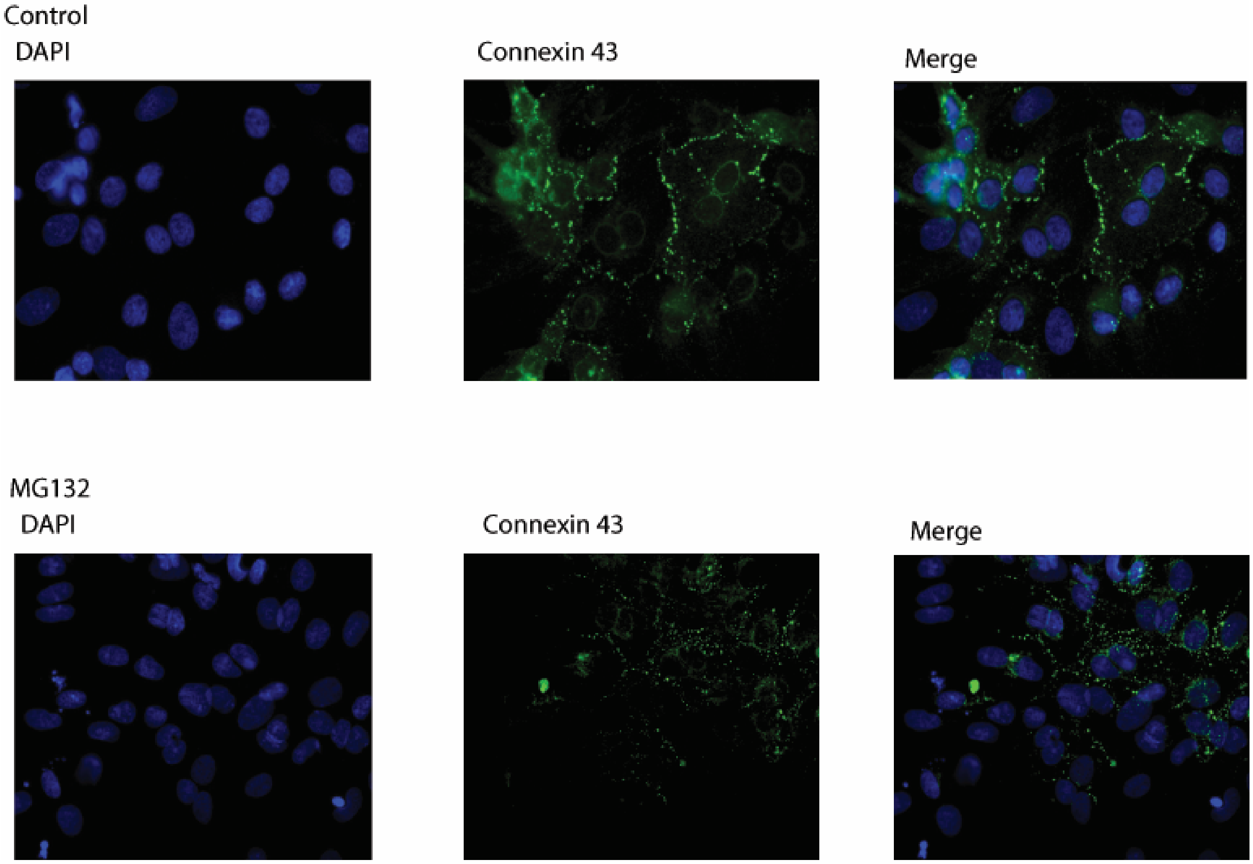

SREP-18-21663A

Figure S1G

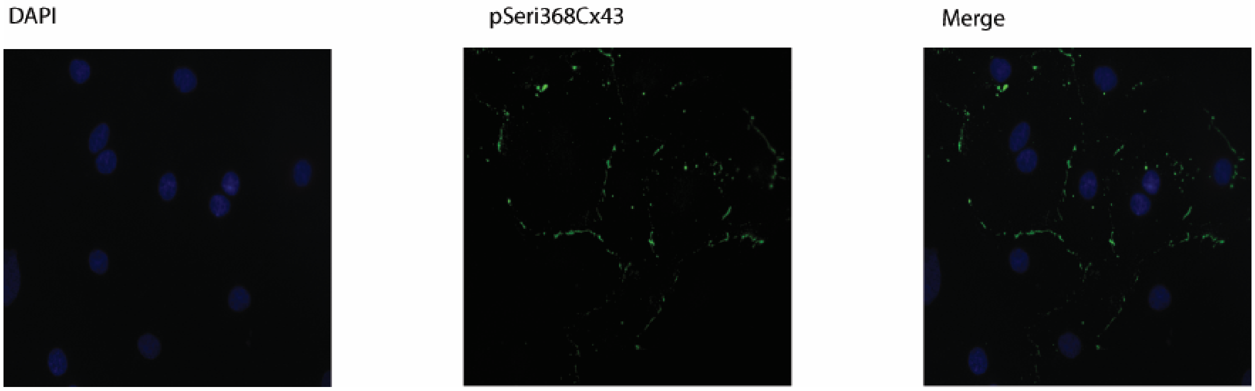

Figure S3

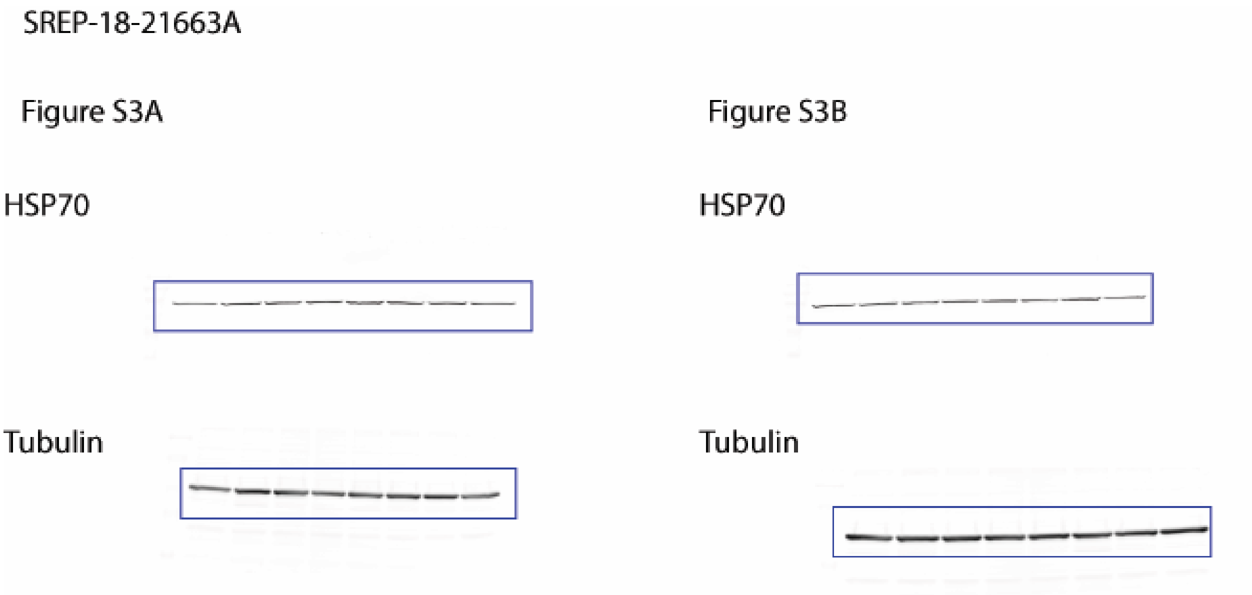
SREP-18-21663A
Figure S3A
Figure S3B
HSP70
HSP70
Tubulin
Tubulin
